# Analyzing an organism’s sensors using Maximum Entropy (MaxEnt) models with bias, variance, and confusion matrices

**DOI:** 10.1371/journal.pone.0342165

**Published:** 2026-08-11

**Authors:** Christopher Wang, Elianna Schimke, Tristan Kako, Aiden Gao, Martina Lamberti, Joost le Feber, Sarah Marzen

**Affiliations:** 1 Department of Natural Sciences, Scripps and Pitzer Colleges, Claremont, California, United States of America; 2 School of Arts and Sciences, Tufts University, Medford, Massachusetts, United States of America; 3 Pioneer Academics, Jenkintown, Pennsylvania, United States of America; 4 Université Claude Bernard Lyon1, CNRS UMR5292, INSERM U1028, Sleep Team, Center for Research in Neuroscience of LYON, Bron, France; 5 Department of Clinical Neurophysiology, University of Twente, Enschede, The Netherlands; 6 Kravis Department of Integrated Sciences, Claremont McKenna College, Claremont, California, United States of America; 7 National Institute for Theory and Mathematics in Biology, Chicago, Illinois, United States of America; University of Oxford, UNITED KINGDOM OF GREAT BRITAIN AND NORTHERN IRELAND

## Abstract

Biological organisms have sensors that communicate information about the environment. Analyzing how well these biological sensors function has usually been done with mutual information between the sensor signal and the environment, but that can be computationally intractable and summarize something quite complex with just a single number. We suggest that alternatively, one may profitably analyze these biosensors using bias and variance or confusion matrices, depending on the kind of environment. Stimulus-dependent Maximum Entropy models are used to develop estimators of the environmental state given the sensor state, and these estimators in turn are then used to calculate either the bias and variance of the estimator or confusion matrices. We focus on several examples to understand the utility of non-information-based analyses: ligand-receptor binding models spanning from genetic regulation to neuronal communication to bacterial chemotaxis and spin-glass Ising models for neural activity in cultured neurons. These new computationally efficient analyses add insight into existing analyses based on mutual information; in particular, mutual information estimates give one number to characterize responses to all environmental inputs, and this analysis method characterizes how sensors respond to each environmental input. Categorical analyses, meanwhile, indicate the presence of memory without much prediction in confusion matrix elements in cultured neural networks, adding to previous understanding from mutual information estimates.

## Introduction

Biological organisms understand their environment and subsequently make informed decisions by using their sensors to encode information about the environment through interaction with external stimuli. This produces behavior that represents memory of past stimuli, prediction of future stimuli, or environmental conditions, posited to be the most important functions guiding how any organism navigates its environment [1,2]. However, because encoding and transmitting information costs energy, sensory systems likely keep only a fraction of information influx from sensors, ideally information which maximizes memorization and predictive capabilities. Given this constraint, we are interested in determining how well biological organisms capture information about their environment through their sensors and indeveloping quantitative metrics for how stimuli are represented by biological systems.

Mutual information (MI) [3], which provides insight into how much one signal’s uncertainty is reduced by knowledge of an associated signal, has traditionally been used to gain a better understanding of the interaction between stimulus and network, such as in Ref [4]. It is more effective at understanding nonlinear correlations than a traditional Pearson correlation coefficient [5], even though the Pearson correlation coefficient is easier to calculate, and has a number of operational definitions that sometimes make it a powerful optimality metric [3]. However, it is often difficult to calculate, with many methods running into undersampling, often with high bias and/or high variance [6], though there have been many attempts to fix these issues [7–9]. Moreover, mutual information ultimately only provides one metric about the system, thus sacrificing subtleties of how the model performs, especially when analyzing categorical variables.

An alternative method for analyzing biological systems and understanding how sensory systems interact with stimuli is via Maximum Entropy models in combination with Maximum Likelihood Estimation (MLE) [10–12], quantified with typical statistics metrics. For ordinal environmental variables, e.g., where states are best considered to be real numbers or collections of real numbers, this method is more computationally efficient than MI, while the metrics it yields — bias and variance — are functions of the ordinal variable, therefore providing much more insight into the system compared to a single value from MI alone. Model performance on categorical variables, where environmental states are best considered to be discrete, meanwhile, are best quantified by confusion matrices. Often, bias and variance are reported as averages over the bias and variance per ordinal input, which need not be the case. In fact, a more fine-grained understanding of a sensor can be obtained from understanding how the bias and variance per ordinal input vary. For a categorical variable with n states, we can retrieve $n^{2}-n$ metrics that help us quantify model performance via off-diagonal matrix elements found in Eq 6, also exceeding the amount of information that MI provides. The extra information gives additional insight into the system, showing what kinds of input the sensor prefers and understands.

In this paper, we utilize the extra information that MaxEnt models provide and apply various versions of the model on both ordinal and categorical variables, quantifying what such models hypothesize about the integrity of environmental encoding through bias, variance, and confusion matrix elements. We start by describing experimental methods, including experimental procedure, estimator development, and statistical analysis in Materials and Methods. In the Results and Discussion sections, this is followed by a discussion on obtaining a certain environmental state *x* given sensor state *s* and presenting metrics for both ligand-receptor binding and memory and prediction in cultured cortical neurons with commentary on observed trends. We wrap up the paper with a summary of key takeaways and directions for future work in the Conclusion section.

## Materials and methods

### Statistical mechanical models

Even though statistical mechanical models are well-founded in biophysics by physical arguments, they can be thought of as stimulus-dependent MaxEnt models. The formulation is encapsulated in biophysics textbooks [13]. Essentially, the program is that you can, like in Refs [14,15], write down states of the system, write down multiplicities, write down energies, and from that get statistical mechanical weights and probabilities corresponding to a biological system. Weights of a state are given by the Boltzmann factor $n\exp(-E/k_{B}T)$, where *n* is the multiplicity (the number of ways the state can exist) and *E* the energy of the state. The partition function *Z* is the sum of all weights, and probabilities are weights normalized by the partition function *Z*. Even though biological systems are not in equilibrium, equilibrium statistical mechanical models nonetheless serve as a good approximation [16].

### Culture preparation

We reanalyzed experimental data that were obtained from in vitro cultures of dissociated rat cortical neurons on multi electrode arrays at University of Twente, Netherlands in a study by Lamberti et al [17]. Below we give a summary of culture preparation, recording setup, and experimental design. Readers interested in further details are encouraged to peruse Ref [17].

Neurons were obtained from newborn rats, dissociated by trypsin treatment and trituration, and then plated on multi electrode arrays (MEAs; Multi Channel Systems, Reutlingen, Germany). MEAs were stored in an incubator, under standard conditions of $36^{\circ }$C, high humidity, and 5% CO_2_ in air. All cultures were grown for at least 3 weeks before experiments started, to allow for culture maturation [18–20]. For experiments cultures were transferred to a recording setup in which standard conditions of $36^{\circ }$C, high humidity, and 5% CO_2_ were maintained. Recording began after a 15 minute accommodation period. At the end of each experiment, cultures were returned to the incubator. To make cultures responsive to light (application of optogenetic stimulation) we virally transfected them with an adeno-associated virus that contained the ChannelRhodopsin-2 gene, driven by the CaMKIIα promoter, which is found exclusively in excitatory neurons.

All surgical and experimental procedures were approved by the Dutch committee on animal use (Centrale Commissie Dierproeven; AVD110002016802) and complied with Dutch and European laws and guidelines.

### Recording setup

To record activity, MEAs were placed into a setup outside the incubator, consisting of a MC1060BC preamplifier and FA60s filter amplifier (both MultiChannelSystems GmbH, Reutlingen, Germany). Signals from the network were recorded by 59 electrodes using a custom-made Lab-View program, at a sampling frequency of 16 kHz per electrode. Acquired analogue signals were band-pass filtered (2^nd^ order Butterworth 0.1–6 kHz) before sampling [17,10]. Action potentials were detected whenever recorded voltages exceeded a threshold, set at 5.5 times the estimated root mean square noise level (ranging 3−5 μV).

First, one hour of spontaneous activity was recorded in all cultures, followed by 20 hours of focal or global stimulation. Focal electrical stimulation was applied through one electrode; for global (optogenetic) stimulation, power LEDs were placed above the MEA. Inter-stimulus intervals (ISIs) were drawn independently and identically from a density distribution designed to produce long-range temporal correlations (fractal renewal process) [21] and read from a pre-generated list.

### Estimate accuracy

In all cultures, action potentials were recorded from 59 electrodes, which included spontaneous activity as well as responses to optogenetic or electrical stimulation. Time stamps of detected action potentials and corresponding electrode numbers were stored and analyzed offline. All cultures were stimulated for 20 hours. Neural activity and the environmental stimulus were then binned by creating successive windows of a certain temporal width Δt of activity, or bins, in which one or more spikes resulted in a 1 in that bin and no spikes resulted in a 0 in that bin. This resulted in 720,000 total bins when assuming a time resolution of Δt=100 ms. Responses to electrical stimulation were typically recorded in the time bin of stimulation and the consecutive bin. Due to the relatively slow opening of ion channels upon light activation, responses to optogenetic stimulation reached their maximum within the two bins following the stimulus bin.

Activity and stimulation in each bin are represented by a 60-element vector (59 electrodes and 1 stimulus), thus creating a 60 by 720,000 binary matrix. This matrix contains mostly zeros, as only about 15,500 of the 720,000 bins (2.15%) contain stimulation, and most of the electrodes are inactive in the majority of bins, particularly when not stimulated.

Due to computational complexities discussed thoroughly in the “Applying the model” subsection of the Results section, a subset (ranging between three and six electrodes) of the prepared binary matrix was analyzed using the Maximum Entropy spin-glass Ising model to estimate $J_{xx},\;J_{xs},\;J_{sx},\;J_{ss}$ in Eq 6 and subsequently to calculate a likelihood ratio *L* (Eq 25) and estimator $\hat{x}(s)$. Finally, $\hat{x}(s)$ was compared to the stimulus row list of the real data *x*. Each match between $\hat{x}(s)$ and *x* was summed and divided by the total number of bins to compute an overall estimate accuracy of the experiment, and a confusion matrix was generated tabulating the errors made in guessing *x*.

### Statistical analysis

The Shapiro-Wilk test was used to confirm normality within treatments. On the condition of normality, *t*-tests were used to determine the significance of differences between the control and stimulated experiments. Data were alternatively analyzed with nonparametric tests if the Shapiro-Wilk test determined a non-normal distribution. Statistical significance was determined using an α-level of 0.05.

## Results

### Stimulus-dependent MaxEnt models as sensor models

In order to proceed, we need p(x∣s), where *x* is the environmental variable and s is the sensor state. This is achieved either through a stimulus-dependent MaxEnt model or through an equilibrium statistical mechanical model that incorporates an environmental variable.

Stimulus-dependent MaxEnt models are not so common, but there are a plethora of procedures for creating equilibrium statistical mechanical models of biological processes. One might wonder if, in fact, these procedures need to be altered for our method. The answer to this is a simple “no”. It is, in fact, common for statistical mechanical models of biological processes to implicitly act as conditional distributions that describe how sensor state relates to environmental signal. For instance, if a statistical mechanical model is made of gene regulation, then the probability of RNA polymerase (RNAP) binding depends on concentrations of transcription factors, which in turn relate to environmental signals such as sugar concentration [14]. This example is made as explicit as possible later. As a result, the typical methodology for generating statistical mechanical models from biological cartoons needs no augmentation for the method proposed in this manuscript to be used.

#### Stimulus-dependent MaxEnt models.

To make a stimulus-dependent MaxEnt model, we depart somewhat from Refs [11,12] and instead make a MaxEnt model of the joint probability distribution of environment *x* and sensor s. These models take the form of an exponential linear model,


$$\begin{array}{c} p_{model}(x,s)=\frac{1}{Z}\exp\left(-E_{\theta }(x,s)\right) \end{array}$$ (1)

where *Z* is the partition function, or the sum of $\exp(-E_{\theta }(x,s))$ over all values of *x* and s, which serves as a normalization factor. Assuming the distribution is constrained and parametrized by a set of parameters θ, we aim to fit the data distribution so that the Kullback-Leibler (KL) divergence between model and data, $D_{KL}[p_{data}||p_{model}]$, is minimized, where


$$\begin{array}{c} D_{\text{KL}}[p_{\text{data}}||p_{\text{model}}]=\sum_{x,s}p_{\text{data}}(x,s)\log\frac{p_{\text{data}}(x,s)}{p_{\text{model}}(x,s)}. \end{array}$$ (2)

This is an alternative formulation of MaxEnt models that differs from the usual treatments in which we aim to make a model that is as unbiased as possible using given information from data, such as in Ref [11]. From the joint MaxEnt model one can derive


$$\begin{array}{c} p_{\text{model}}(x|s)=\frac{p_{\text{model}}(x,s)}{p_{\text{model}}(s)}. \end{array}$$ (3)

It turns out that for the analysis described later, we will not need $p_{\text{model}}(s)$. As such, the joint MaxEnt model is as good as the stimulus-dependent MaxEnt model [12] for sensor analysis. If instead $p_{\text{model}}(s|x)$ is obtained, p(x∣s) can be approximated using Bayes rule, as we describe later in Eq 8.

Here we give a brief summary of Ref [11] to illustrate the typical MaxEnt model. Neuronal activity between time *t* and t+Δt is encoded by a binary vector σ, where Δt=100 ms. If neuron *i* fires in that time frame, $\sigma_{i}$ is set to 1, otherwise, $\sigma_{i}=0$. After construction of all binary vectors that occur during a long term recording, their probability distributions are modeled as


$$\begin{array}{c} p_{\text{model}}(\sigma )=\frac{1}{Z}\exp\left(-\theta \cdot \sigma +\sigma^{⊤}J\sigma \right) \end{array}$$ (4)

where *Z*, the partition function, is a normalization factor:


$$\begin{array}{c} Z=\sum_{\sigma }\exp\left(-\theta \cdot \sigma +\sigma^{⊤}J\sigma \right). \end{array}$$ (5)

In Eq 4, θ is a vector that represents the propensity of neurons to fire. *J* is symmetric by definition with zeroes on the diagonal (as those are explicitly represented by θ), and represents first order interactions between neurons.

To adapt such a model for our use case, we fit a joint MaxEnt model to the stimulus *x* and network activity s, $p_{\text{model}}(x,s)$. This is done by concatenating the network activity vector s and stimulus *x* into a binary vector σ whose first elements describe neural activity and whose last element indicates whether or not there was a stimulus. We can “time-shift” *x* from s to simulate memory or prediction, as demonstrated in Ref [17]. Notably, this procedure allows the data to speak directly to temporal correlations between sensor state and environmental signal with an explicit time lag, rather than requiring the user to understand all temporal correlations and autocorrelations.

We thus consider the vector $\sigma =(\begin{array}{c} x\\ s \end{array})$ and maximize the entropy of p(σ) while constraining the mean of σ, ⟨σ⟩, and the second moment, ⟨σ·σ⟩. This leads to the joint MaxEnt model


$$\begin{array}{c} p_{\text{model}}(x,s)=\frac{1}{Z}\exp\left((\begin{array}{cc} x & s \end{array})(\begin{array}{cc} J_{xx} & J_{xs}\\ J_{sx} & J_{ss} \end{array})(\begin{array}{c} x\\ s \end{array})\right). \end{array}$$ (6)

Here, *J* is decomposed into four block matrices. Assuming a system size of *n*, $J_{xx}$ is a scalar interpreted as the stimulus firing propensity, $J_{sx}=J_{xs}^{⊤}$ are (n−1)-dimensional vectors that represent stimulus-network couplings, and $J_{ss}$ is an (n−1)×(n−1) matrix describing the couplings between network elements. Eq 6 incorporates both moment constraints: because σ is a binary vector, each element satisfies $\sigma_{i}^{2}=\sigma_{i}$, and therefore the diagonal of *J* accounts for the mean constraint by doubling as a magnetic field analogue from the Ising model. Meanwhile, the off-diagonal elements of *J* handle the second moment constraints.

To account for class imbalance (the stimulus is much more likely to be inactive than active), we weighted the KL divergence appropriately such that inaccuracies in guessing activity were penalized proportionately. Moreover, we used the scipy.optimize.minimize module for model fitting with a custom-designed KL function to account for class imbalance. In the minimization process, we used the L-BFGS-B optimizer method and ran a maximum of 200 iterations or until the difference between successive iterations was less than a set tolerance of 10^−7^. Specifics of implementation can be found in the Data Availability Statement.

#### Statistical mechanical models.

**Fig 1:**
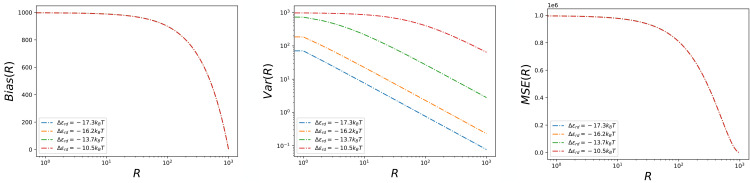
Bias, variance, and MSE of the lacR estimator based on whether or not RNAP is bound. At left, bias as a function of the number of lacR molecules *R*. In the middle, variance as a function of the number of lacR molecules *R*. At right, mean-squared error (MSE) as a function of the number of lacR molecules *R*. The energy of lacR binding is varied and $N_{NS}=5\times 10^{6}$ chosen as in Ref [14].

Statistical mechanical models are, in some way, examples of stimulus-dependent MaxEnt models. An example is shown in Fig 1 for the lac repressor genetic regulatory circuit described in Ref [14]. The result of this statistical mechanical calculation is the conditional probability of RNAP binding to the promoter region given that there is a certain number of lac repressor molecules. We may use this variable as the environmental variable due to the intimate relationship between it and lactose molecules: as lactose molecules — the true environmental variable — appear, they bind to lac repressor molecules in a chemical reaction that leads to a certain concentration of free lac repressor molecules being a direct readout of the concentration of lactose molecules. The output of the statistical mechanical model [14] is the following model for $p_{\text{model}}(s|x)$, where *s* is whether or not RNAP is bound and *x* is the number of Lac repressor (lacR) tetramers:


$$\begin{array}{c} p_{\text{model}}(s|x)=\frac{p_{0}}{1+p_{0}+\frac{x}{N_{\text{NS}}}\exp(-\beta \Delta \epsilon_{\text{rd}})}. \end{array}$$ (7)

In the case of a single sensor, we may switch from the boldfaced s to *s* to denote sensor state. Here, *p*_0_ is the number of RNAP molecules, *P*, divided by the number of nonspecific DNA sites, $N_{NS}$, with values of $p_{0}\approx 10^{-3}$ and $N_{\text{NS}}\approx 5\times 10^{6}$, and $\Delta \epsilon_{\text{rd}}$ is the repressor-DNA binding energy difference, which adopts a range of values that can be tuned in experiments [14]. Finally, $\beta =1/k_{B}T$, the inverse temperature.

With p(s∣x), we can find p(x∣s) via Bayes’ theorem:


$$\begin{array}{c} p(x|s)=\frac{p(s|x)p(x)}{p(s)}. \end{array}$$ (8)

We will often assume a uniform input concentration *p*(*x*), as it is the most unbiased model of the environment with no constraints. If naturalistic statistics of the environmental variable are known, as is the case for naturalistic scenes or videos or auditory signals, then *p*(*x*) can be replaced with a model of the environmental variable. The sensor marginal distribution *p*(*s*) will not be explicitly needed, as we will see in the next subsection.

Another example of a statistical mechanical model that describes everything from bacterial chemotactic receptors to nicotinic acetylcholine (nACh) receptors is the Monod-Wyman-Changeux (MWC) model [15], according to which the probability of a receptor with *n* sites being active is


$$\begin{array}{c} p_{\text{active}}(x)=\frac{{\left(1+\frac{x}{K_{d}^{\left(A\right)}}\right)}^{n}}{{\left(1+\frac{x}{K_{d}^{\left(A\right)}}\right)}^{n}+L{\left(1+\frac{x}{K_{d}^{\left(I\right)}}\right)}^{n}} \end{array}$$ (9)

for dissociation constants $K_{d}^{\left(A\right)}$ and $K_{d}^{\left(I\right)}$, ligand concentration *x*, and a parameter *L* that governs the energetic difference between the active and inactive state. This is alternatively p(s∣x), where *s* is whether or not the MWC molecule is active. When applied to bacterial chemotaxis, there are two types of binding sites, and the model generalizes to


$$\begin{array}{c} p_{\text{active}}(x)=\frac{{\left(1+\frac{x}{K_{d}^{\left(A,1\right)}}\right)}^{m}{\left(1+\frac{x}{K_{d}^{\left(A,2\right)}}\right)}^{n}}{{\left(1+\frac{x}{K_{d}^{\left(A,1\right)}}\right)}^{m}{\left(1+\frac{x}{K_{d}^{\left(A,2\right)}}\right)}^{n}+L{\left(1+\frac{x}{K_{d}^{\left(I,1\right)}}\right)}^{m}{\left(1+\frac{x}{K_{d}^{\left(I,2\right)}}\right)}^{n}}. \end{array}$$ (10)

### Analyzable estimators of the environment from sensor models

Once we have $p_{\text{model}}(x|s)$, we utilize Maximum a Posteriori (MAP) estimation to form an estimator of the environment from the sensor state, mathematically represented as


$$\begin{array}{c} \hat{x}(s)=\underset{x}{\text{argmax}}\;p_{\text{model}}(x|s), \end{array}$$ (11)

where $\underset{x}{\text{argmax}}\;f(x)$ yields the argument *x* that maximizes *f*(*x*). If we have a joint MaxEnt model, we can find this as


$$\begin{array}{c} \hat{x}(s)=\underset{x}{\text{argmax}}\;\frac{p_{\text{model}}(x,s)}{p_{\text{model}}(s)}=\underset{x}{\text{argmax}}\;p_{\text{model}}(x,s). \end{array}$$ (12)

If instead we have $p_{\text{model}}(s|x)$ as with the statistical mechanical models, we can find the estimator as


$$\begin{array}{c} \hat{x}(s)=\underset{x}{\text{argmax}}\;\frac{p_{\text{model}}(s|x)\;p(x)}{p(s)}=\underset{x}{\text{argmax}}\;p_{\text{model}}(s|x)\;p(x). \end{array}$$ (13)

We can approximate *p*(*x*) from data or from another model. For our particular neural system, we use *p*(*x*)  =  *p*_data_(*x*). We could instead use an unbiased model of the environment, making *p*(*x*) uniform, leading to


$$\begin{array}{c} \hat{x}(s)=\underset{x}{\text{argmax}}\;p_{\text{model}}(s|x). \end{array}$$ (14)

This corresponds to Maximum Likelihood Estimation. In making these estimates, we imagine a downstream region of the brain or organism finding the sensor state and trying to invert it to find an estimate of the environment. This estimate of the environmental state can then be used to inform action policy.

### Bias, variance, and mean-squared error with ordered environmental variables

If the environmental variable is ordinal, then we can form bias, variance, and mean-squared error estimates of the environmental variable as follows:


$$\begin{array}{ccc} \text{Bias}(x) & = & \langle \hat{x}(s)\rangle_{p_{\text{model}}(s|x)}-x \end{array}$$ (15)

$$\begin{array}{ccc} \text{Var}(x) & = & \langle {\left(\hat{x}(s)-\langle \hat{x}(s)\rangle_{p_{\text{model}}(s|x)}\right)}^{2}\rangle_{p_{\text{model}}(s|x)} \end{array}$$ (16)

$$\begin{array}{ccc} \text{MSE}(x) & = & \langle {\left(\hat{x}(s)-x\right)}^{2}\rangle_{p_{\text{model}}(s|x)}. \end{array}$$ (17)

We can use MSE(*x*) = Bias(*x*)^2^ + Var(*x*) to calculate MSE easily. Bias, variance, and MSE of an estimator are all indicators of the estimator’s quality. Bias indicates the accuracy, while variance indicates its precision and MSE indicates its overall quality.

We are unable in most cases to report mutual informations because data is not available on ligand concentration profiles that are likely to be seen by the receptors. However, given the receptor’s biophysics as specified by the statistical mechanical model, there is a maximum mutual information that can be attained if the probability distribution over ligand concentrations is of a certain form. This maximum mutual information is the channel capacity, and the probability distribution that achieves this channel capacity is the capacity-achieving distribution. Thus, to compare with mutual information analyses, we calculate channel capacity and report both the channel capacity and the capacity-achieving distribution. Roughly speaking, one might say that receptors “like” the ligand concentrations for which the capacity-achieving probability is large, although this statement is unsubstantiated and hand-wavy at best. We therefore can compare the capacity-achieving distribution to the mean-squared error curves in order to understand the subtle differences between a mutual information analysis and our new analyses. Mean-squared error will be small if our analysis reveals that a particular ligand concentration is “liked” by the receptor, while the capacity-achieving distribution will be high if a mutual information analysis reveals that a certain ligand concentration is preferred. Standard code is available for calculating the capacity-achieving distribution and the channel capacity via the Blahut-Arimoto algorithm [22] in the GitHub repository.

**Fig 2:**
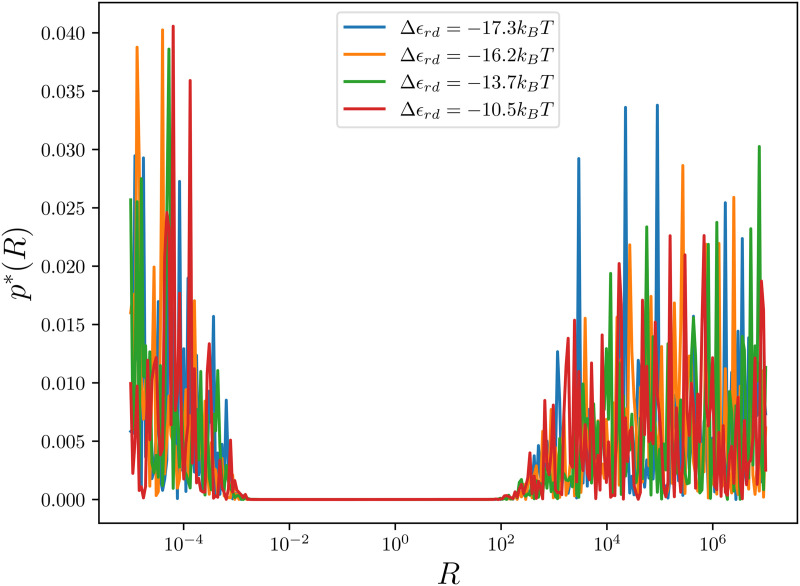
A channel capacity analysis of repressors in gene regulation reveals preferences for low and high concentrations. Plotted are the optimal, capacity-achieving distributions over repressor values, $p^{*}(R)$. For all $\Delta \epsilon_{rd}$ values, channel capacity was found to be *C* = 0.00051 bits. The energy of lacR binding is varied and $N_{NS}=5\times 10^{6}$ chosen as in Ref [14]. 10,000 iterations of the Blahut-Arimoto algorithm were used to find channel capacity and the capacity-achieving distribution.

To illustrate, bias, variance, and MSE are calculated for the simple genetic regulatory circuit in Ref [14] in Fig 1 below. We find that when RNAP is bound, we should estimate that there are no lacR molecules in the environment, and that when RNAP is not bound, we should estimate that there are as many lacR molecules in the environment as possible, regardless of binding energy $\Delta \epsilon_{\text{rd}}$. Typically, RNAP is not bound. As such, this estimator leads to bias estimates that are large when the number of lacR molecules is small and MSE estimates that are large when the number of lacR molecules is small. The MSE is mostly dominated by the bias. The variance (estimator noisiness) depends more intricately on the details of the binding energy of lacR to the DNA site. When the binding energy is less favorable, we see a noisier estimator of lacR molecules, as the probability of RNAP binding is higher overall. A channel capacity analysis, according to Fig 2, would indicate incorrectly that this gene regulatory circuit prefers low and high repressor concentrations rather than just the large repressor concentrations indicated by our new method.

**Fig 3:**
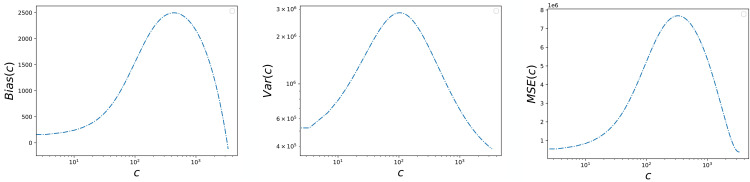
Bias, variance, and MSE of the ACh concentration estimator based on whether or not nACh is in the active (open) state. At left, bias as a function of the ACh concentration *c*. In the middle, variance as a function of the ACh concentration *c*. At right, mean-squared error (MSE) as a function of the ACh concentration *c*. Parameters are taken from Ref [23].

**Fig 4:**
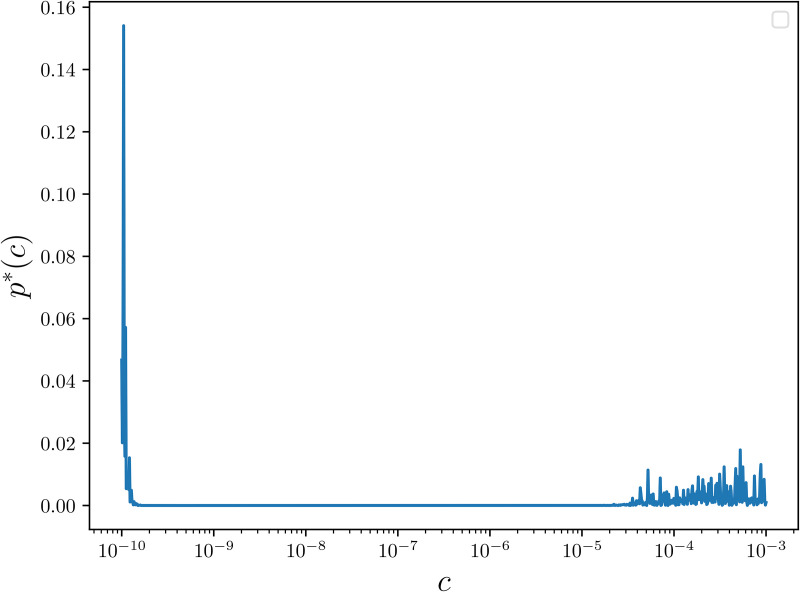
A channel capacity analysis of nACh receptors reveals preferences for very low and very high ACh concentrations. Plotted are the optimal, capacity-achieving distributions over ACh concentrations, $p^{*}(c)$. Channel capacity was found to be *C* = 0.67 bits. Parameters are taken from Ref [23]. 10,000 iterations of the Blahut-Arimoto algorithm were used to find channel capacity and the capacity-achieving distribution.

Bias, variance, and MSE are also calculated for the nACh molecule using the Monod-Wyman-Changeux model from parameters in Ref [23] and shown in Fig 3. The estimator gives a maximal acetylcholine (ACh) concentration estimate when the nACh receptor is active (open) and an ACh concentration estimate of 0 M when the nACh receptor is inactive (closed). From this, bias is maximal at middling concentrations. Variance is maximized when the probability of the receptor being active is 1/2. Together, these determine a mean-squared error estimate that is large at middling concentrations, due to large bias and variance. Unlike the channel capacity calculation in Refs [15,24], we do not need to assume that the organism controls the environment (*p*([ACh])), and we also gain insight into which environmental variables are well-represented — the analogue of understanding the optimal *p*([ACh]) that maximizes mutual information in Refs [15,24], which shows peaks at low and high concentrations. This time, a channel capacity analysis, according to Fig 4, indicates correctly that this receptor prefers low and high ACh concentrations both, although our new method finds that low and high ACh concentrations are near-equally appreciated.

**Fig 5:**
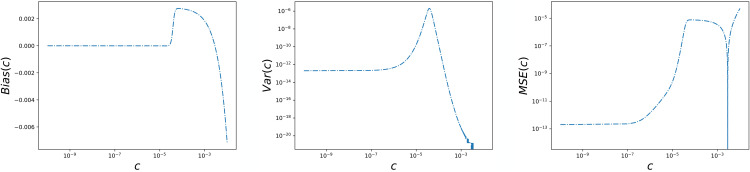
Bias, variance, and MSE of the chemoattractant concentration based on whether or not the receptors are in the active state. At left, bias as a function of the chemoattractant concentration *c*. In the middle, variance as a function of the chemoattractant concentration *c*. At right, mean-squared error (MSE) as a function of the chemoattractant concentration *c*. Parameters are taken from Ref [25] with *m* = 0 (*m* is the average receptor methylation level in the receptor complex) and *c*_0_ = 0 (*c*_0_ is the ambient concentration of the attractant chemical).

**Fig 6:**
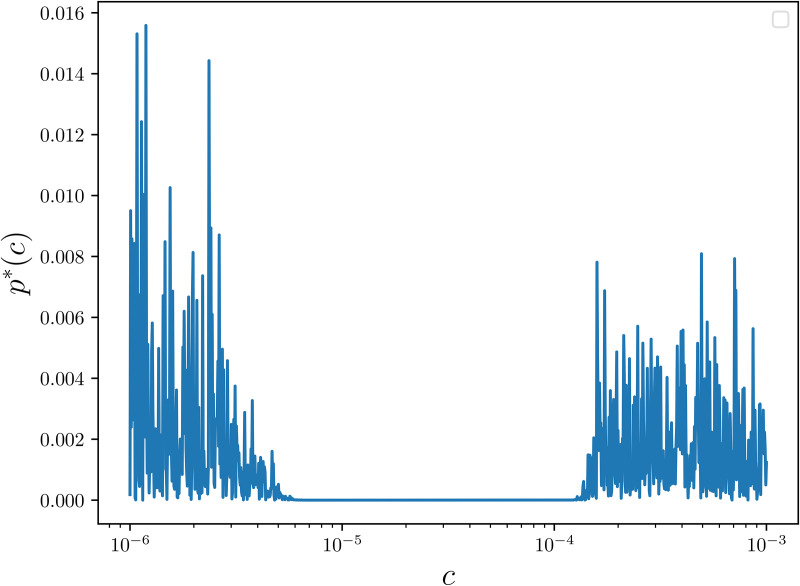
A channel capacity analysis of bacterial chemotactic receptors reveals preferences for very low and very high chemoattractant concentrations. Plotted are the optimal, capacity-achieving distributions over chemoattractant concentrations, $p^{*}(c)$. Channel capacity was found to be *C* = 1.0 bits to within $2\times 10^{-6}$. Parameters are taken from Ref [25] with *m* = 0 and *c*_0_ = 0. 200,000 iterations of the Blahut-Arimoto algorithm were used to find channel capacity and the capacity-achieving distribution.

Bias, variance, and MSE are also calculated for the bacterial chemotactic receptors using the Monod-Wyman-Changeux model from parameters in Ref [25] and shown in Fig 5. The estimator gives a minimal chemoattractant concentration estimate when the bacterial chemotactic receptors are inactive and a high but not maximal chemoattractant concentration estimate when the receptors are active. Again, from this, bias is maximal at middling concentrations and at higher concentrations than the maximal concentration estimate. Again, variance is maximized when the probability of the receptor being active is 1/2. Together, these determine a mean-squared error estimate that is small at smaller concentrations and larger at larger concentrations, with a minimum at the maximal concentration estimate. Unlike a channel capacity calculation, we do not need to assume that the bacterium controls the environment (*p*(*x*)), and we also gain insight into which environmental variables are well-represented– the analogue of understanding the optimal *p*(*x*) that maximizes mutual information, which shows valleys when *variance* of the estimator is low. A channel capacity analysis, according to Fig 6, indicates incorrectly that this receptor merely prefers low and high chemoattractant concentrations when in fact the receptor appreciates mostly low chemoattractant concentrations, as might be expected from previous analyses [26]. This calculation reveals not only the noisiness of the estimator but also the accuracy of the estimator from the bias, and therefore provides more detail than the corresponding channel capacity calculation.

To showcase new insights that can be obtained using this analysis method, we turn to asking for the benefit or downside of cooperative sensors. Previously, cooperativity in sensors was shown to aid information processing [15,24,27]. Yet, a simple mutual information analysis says independent is better: $I[s_{1},s_{2};x]=I[s_{1};x]+I[s_{2};x|s_{1}]$ is maximized if $s_{1},s_{2}$ are conditionally independent given *x*. Does cooperativity in a two-state Adair model, described below, do anything to information processing as measured by bias, variance, and MSE? We have:


$$\begin{array}{ccc} p(s_{1}=0,s_{2}=0|x) & = & \frac{1}{Z} \end{array}$$ (18)

$$\begin{array}{ccc} p(s_{1}=1,s_{2}=0|x) & = & \frac{x/K}{Z} \end{array}$$ (19)

$$\begin{array}{ccc} p(s_{1}=0,s_{2}=1|x) & = & \frac{x/K}{Z} \end{array}$$ (20)

$$\begin{array}{ccc} p(s_{1}=1,s_{2}=1|x) & = & e^{-\beta \Delta \epsilon }{\left(x/K\right)}^{2}/Z, \end{array}$$ (21)

$$\begin{array}{ccc} \text{where }Z & = & 1+2(x/K)+e^{-\beta \Delta \epsilon }{\left(x/K\right)}^{2}. \end{array}$$ (22)

**Fig 7:**
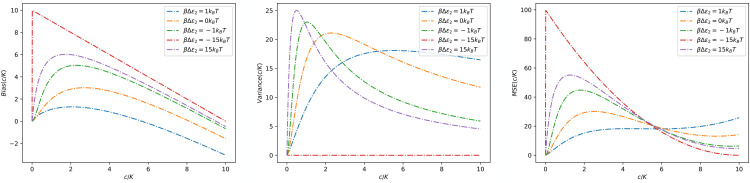
The bias, variance, and MSE of a sensor with varying degrees of cooperativity. At left, bias as a function of the chemoattractant concentration *c*. In the middle, variance as a function of the chemoattractant concentration *c*. At right, mean-squared error (MSE) as a function of the chemoattractant concentration *c*. A range of cooperativities based on varying $\beta \Delta \epsilon_{2}$ are shown.

To briefly contextualize: *x* is the ligand concentration, *K* is the dissociation constant, Δϵ is the cooperativity energy, and $\beta =1/k_{B}T$. If we look at MSE, there is a range of ligand concentrations for which cooperativity improves performance (lower MSE) and a range of ligand concentrations for which it does not. The answer to the question of whether or not cooperativity benefits sensor performance depends on variables such as what kinds of ligand concentrations the sensor is likely to see. See Fig 7.

### Confusion matrices with memory and prediction in cultured neurons

Because behavior in all cultures was monitored by 59 electrodes [17], whose signals were used to construct a prediction about the activity of the optogenetic or electrical stimulus, our estimator ultimately makes a categorical prediction. Therefore, it is most productive to use confusion matrices to quantify model performance. Since the estimator guesses the activity or inactivity of the stimulus, it is binary, and thus we may avoid calculating the partition function and instead develop a likelihood ratio


$$\begin{array}{ccc} L(s) & = & \frac{p(x=1|s)}{p(x=0|s)} \end{array}$$ (23)

$$\begin{array}{ccc} & = & \frac{\exp\left((\begin{array}{cc} 1 & s^{⊤} \end{array})(\begin{array}{cc} J_{xx} & J_{xs}\\ J_{sx} & J_{ss} \end{array})(\begin{array}{c} 1\\ s \end{array})\right)/Z}{\exp\left((\begin{array}{cc} 0 & s^{⊤} \end{array})(\begin{array}{cc} J_{xx} & J_{xs}\\ J_{sx} & J_{ss} \end{array})(\begin{array}{c} 0\\ s \end{array})\right)/Z} \end{array}$$ (24)

$$\begin{array}{ccc} & = & \exp\left(J_{xx}+J_{xs}s+s^{⊤}J_{sx}\right). \end{array}$$ (25)

If *L*(*s*) > 1, then $\hat{x}(s)=1$; otherwise $\hat{x}(s)=0$.

#### Creating controls.

When assuming a time bin of Δt=100 ms, approximately 97.85% of all entries in the stimulus electrode were inactive, thus positively biasing the accuracy of stimulus estimates $\hat{x}(s)$. To assess this bias and its effects on confusion matrix outputs, control data were generated and analyzed using the MaxEnt model. First, assuming no correlation between any of the neurons and a random firing rate, completely random data are generated, and the estimator should not be biased into guessing either activity or inactivity over the other. Thus, the estimator should predict correctly half the time, resulting in a confusion matrix as in Table 1.

**Table 1:** The confusion matrix generated for completely random data. Given this assumption, the model is expected to correctly predict both electrode activity (true positive rate, bottom right entry) and inactivity (true negative rate, top left entry) half of the time. Incorrect predictions are documented in off-diagonal entries, and each column must sum to 100% to account for all data.

	Actual 0	Actual 1
Predicted 0	50%	50%
Predicted 1	50%	50%

Next, we relax the assumption that none of the neurons are correlated but maintain the assumption that the stimulus and network are uncorrelated. Under this constraint, the off-diagonal terms in the *J* matrix — related to the correlation strengths between neurons [10,11] — do not contribute to the likelihood ratio, which thus is dominated by the activity propensity of the stimulus. To generate simulated data under these conditions, we constructed a purely diagonal *J* matrix with negative coefficients on the diagonal to reflect the fact that electrodes are more likely to be inactive than active, then analyzed it using our model. The model defaults to estimating exclusively inactivity for the duration of our experiment, thus achieving 97.85% baseline accuracy at a time bin of Δt=100 ms.

#### Applying the model.

**Fig 8:**
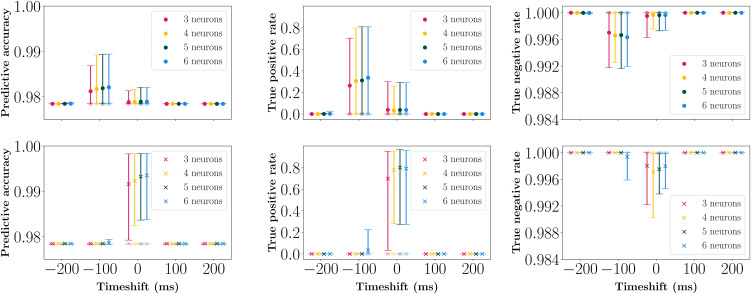
Predictive accuracy, true positive rate, and true negative rate for baseline and model estimators with 95% confidence intervals. Colors correspond to varying neuron subsystem sizes used for determining the *J* matrix. Circular markers denote optogenetic (*n* = 10) stimulation (top row), crossed markers denote electrical (*n* = 8) stimulation (bottom row). Plots with varying system sizes at the same time-shift are staggered to enhance visibility of markers and error bars.

To develop an estimator, we used the Maximum Entropy spin-glass Ising model. Because this model requires calculation of the partition function, we chose a subset of the 60 neurons to construct the network vector *s*. Calculating the full partition function for *n* neurons requires summing over all $2^{n}$ configurations of the system and, for our case of 60 neurons, is not computationally feasible. Therefore, it is worth investigating how much information can be captured with a subset of the entire population. To choose a subset, we greedily add neurons based on how much they contribute to improving predictive accuracy of our estimator, similar to the algorithm used in Ref [17]. Starting with just the stimulus neuron, we first exhaustively iterate over a subset of two neurons from the pool of 59 neurons, choosing the neuron that best improves predictive accuracy. This neuron is appended to the subset, fixed in place, and an exhaustive search is now done with a three-neuron subset by examining each of the remaining 58 options. This process is repeated until the desired subset size is obtained. With this method, subsystem sizes were chosen from three to six neurons. The MaxEnt model was then fitted to the data, and the confusion matrix elements characterizing the estimator’s performance are shown in Fig 8.

This model can also be applied after time-shifting the stimulus *x* relative to the network activity *s*, allowing us to assess prediction and memory confusion matrices. Incorporating a positive-signed time-shift is physically interpreted as assessing the system’s prediction capability, while a negative-signed time-shift assesses the system’s memory capability [17]. Time-shifting was performed out to ±200 ms; larger time-shifts relegated model performance to baseline. Time-shifting implicitly takes into account temporal autocorrelations in the network state and the stimulus state, as described in Ref [17]. If there are temporal autocorrelations in the network activity, they will automatically allow for potentially reduced correlations between past or future stimulus activity and present network activity. One way to think about this is to notice that we are essentially marginalizing a probability distribution over successive network states and successive stimulus states in order to retain just the probability distribution over past or future stimulus states and the present network state; but this can be just as well accomplished simply by fitting a Maximum Entropy model to past or future stimulus states and present network states without first fitting to successive network states and then marginalizing.

Statistics of model metrics over experiments were explored to compare optogenetic and electrical trials. Only two of the time-shifts in Fig 8, −100 and 0 ms, are relevant: optogenetic and electrical predictive accuracies are not at all distinguishable at other time-shifts. At a time-shift of 0 ms and a subsystem size of six neurons, the model for electrical stimulation was normally distributed. With α=0.05, performing a Shapiro-Wilk normality test on electrically stimulated samples yielded a *p*-value of *p* = 0.0998. However, the same test on optogenetic samples yielded a *p*-value of $p=1.13\cdot 10^{-7}$, indicating non-normality. Since the optogenetic stimulation samples are not normally distributed, to compare the location parameters of electrical and optogenetic stimulation, we first check that each distribution is distinct via the Mann-Whitney *U* test, which is confirmed with a *p*-value of $p=6.16\cdot 10^{-4}$. Using the Hodges-Lehmann estimator, we then find a 1.71% higher predictive accuracy in electrically stimulated samples compared to optogenetically stimulated samples. This is statistically significant even with a Bonferroni correction for multiple comparisons so that the correct significance level is α=0.025. Considering baseline accuracy already lies at about 97.85%, this improvement is equivalent to a fivefold decrease in error rates. We can interpret this as follows: a locally concentrated electrical pulse interacts strongly with a subset of the neurons present, while light pulses interact weakly with the whole system; as such, it is significantly easier to predict behavior in electrical stimulation. Meanwhile, there was no statistically significant difference between globally stimulated cultures and control trials (*p* = 0.319 using the Mann-Whitney *U* test).

Repeating this statistical analysis at −100 ms and again at six neurons, model predictive accuracy outputs were normally distributed for optogenetic stimulation but not for electrical stimulation: Shapiro-Wilk tests revealed $p=1.26\cdot 10^{-4}$ and *p* = 0.0627 for electrical and optogenetic stimulation, respectively. The Mann-Whitney *U* test applied on the optogenetic and electrical model samples indicates similar distributions with *p* = 0.122, but optogenetic samples show a statistically significant difference from baseline with *p* = 0.0214 using Welch’s *t*-test. Interestingly, electrical model samples are almost identical *t*o baseline, with a Mann-Whitney *U* test *p* value of *p* = 1.

Examining electrical stimulation, model performance most significantly improves over the baseline when there is no time-shift and a subset size of six neurons with a mean model predictive accuracy at 99.35% and true positive rate of 79.05%. Simultaneously, zero time-shift also presents the most significant drop in true negative rates, with correct predictions occurring 99.80% of the time as opposed to a baseline of 100%. Fig 8 shows an asymmetry in time-shifting 100 ms, with an incremental improvement in model predictive accuracy compared to baseline for −100 ms and no improvement in the positive direction.

Applying the model to optogenetic stimulation experiments yields most significant improvements at −100 ms time-shift and six neurons, with a mean model predictive accuracy of 98.21%, true positive rate of 33.64%, and true negative rate of 99.63%. At 0 ms time-shift, marginal improvements in true positive rate are observed (about 3.74%), but the decrease in true negative rate negates model predictive accuracy to be comparable to baseline.

Fig 1d in Ref [17] provides more insight into the observed trends. Mean number of spikes is highest immediately following stimulus activity in electrical stimulation, with a vanishing minority of spikes occurring between 100 and 200 ms post-activity. This translates to the system’s memory of the stimulus being strongest right after stimulus activity, which shows up as a significant improvement in model predictive accuracy at 0 ms time-shift. The distribution in electrode spikes for optogenetic stimulation, however, is notably wider because the light pulse itself lasts about 100 ms. Therefore, the mean number of spikes is densest at around 100 ms post-activity. Network electrodes for optogenetic stimulation also do not start spiking until several milliseconds post-activity as opposed to almost immediately for their electrical counterpart, which further accounts for the drastic observed difference in predictive accuracy at 0 ms time-shift.

Varying electrode subset sizes also reveals interesting behavior. Remarkably, even selecting the three network electrodes that contribute most greatly to the predictive accuracy captures the majority of possible improvements, evidenced by increasingly marginal improvements with a larger system size.

Moreover, regardless of stimulation type, none of the systems explicitly displayed detectable predictive behavior, since at any time-shift +100 ms and beyond, all model confusion matrix metrics were identical to baseline. Although we see nonzero predictive mutual information (MI_future_) in Fig 3 of Ref [17], the mutual information relating to predictive capability is more than an order of magnitude smaller than the mutual information relating to memory capability (MI_future_ compared to MI_past_), with the largest MI_future_ on the order of 10^−3^ and the largest MI_past_ on the order of 10^−1^. The comparatively small magnitude of MI_future_ means that the signal may never be strong enough to trigger the threshold of activity in the likelihood estimator of our MaxEnt model, resulting in model performance equivalent to baseline in the regime of positive time-shift. Meanwhile, there is enough mutual information encoded in MI_past_ to trigger improvements above baseline, which is indicated by improved predictive accuracy in our model. The confusion matrix teases out more details with the addition of both true positive rates and true negative rates than the mutual information, indicating how often the network mistakes activity for silence and vice versa.

To understand the effects of time resolution on model metrics, we additionally ran the MaxEnt model on our experiments while varying time resolution, using time bins of Δt∈{25,50,100,200} ms. We used a subsystem size of six electrodes at 0 ms time-shift for electrical stimulation and −100 ms time-shift for optogenetic stimulation to best investigate how each confusion matrix metric varied with respect to experimental time resolution.

**Fig 9:**
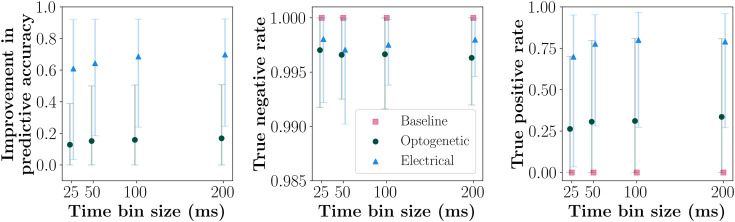
Improvement in predictive accuracy relative to baseline (where 1.0 represents perfect predictive accuracy), true positive rate, and true negative rate for baseline and model estimators with 95% confidence intervals. Colors and marker types correspond to stimulation type (red is baseline) used for determining the *J* matrix. Circular markers denote optogenetic (*n* = 10) stimulation and constitute the first row, while triangular markers denote electrical (*n* = 8) stimulation and constitute the second row. Plots with varying stimulation type at the same time resolution are staggered to enhance visibility of markers and error bars.

Fig 9 does not show a statistically significant improvement in predictive accuracy, true negative rate, or true positive rate against baseline with respect to time resolution coarse-graining. Although there are marginal trends in the mean true positive rate, the confidence intervals have so much overlap that we cannot identify any trends with statistical significance; altering time bin size does not reliably change any of the metrics investigated in Fig 9.

**Fig 10:**
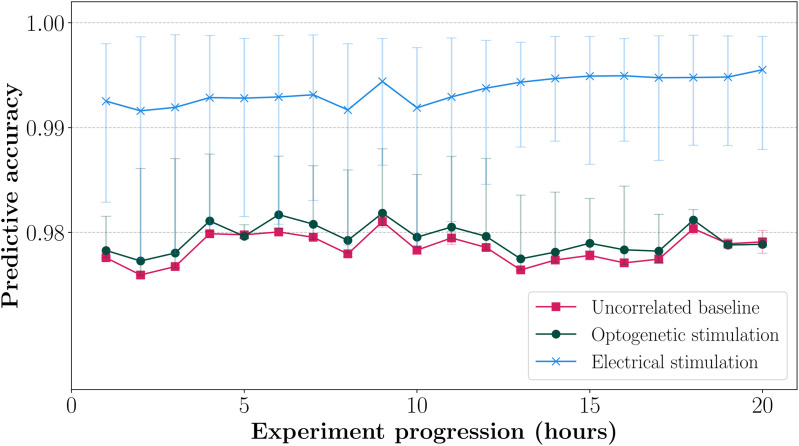
Hour-by-hour mean MaxEnt model predictive accuracy for optogenetic (*n* = 10) and electrical (*n* = 8) stimulation experiments for the experimental duration (20 hours) relative to an uncorrelated baseline estimate. Accuracy was determined with a subsystem of six electrodes at 0 ms time-shift. Error bars represent a 95% confidence interval.

Finally, experiments were decomposed into hour-by-hour segments to investigate whether there were incremental differences throughout the duration of stimulation. Inspecting Fig 10, regardless of stimulation type, there was slight fluctuation from hour to hour but no significant increase or decrease of prediction ability over the course of a 20 hour experiment. The observed high correlation between uncorrelated baseline and global stimulation in Fig 10, while remarkable, is explained by the lack of statistical significance between globally stimulated cultures and control trials. Such results, combined with no observable predictive capability, suggest that the network population may not learn the stimulus but nonetheless responds to stimulus activity in a stimulus-response manner via reverberation of the stimulus around the network.

### Convergence properties of new biosensor metrics

Maximum Entropy parameters are in fact maximum likelihood estimators in that they best fit exponential linear models to data. Because maximum likelihood estimators are consistent, we find that the estimators here (functions of a maximum likelihood estimator) are consistent. Also, maximum likelihood estimators are asymptotically normal with variance that goes as 1/*N* and a bias that does at worst the same [28]. Here we offer a sketch of a proof for how that translates to convergence of the estimators of bias, variance, mean-squared error, and confusion matrices. In the limit that the amount of data is large, 1/*N* is quite small, and we are justified in making a small noise approximation in which statistics are roughly Gaussian. In particular, the variances of confusion matrix elements or bias, variance, and mean-squared error can be said to vary linearly with the variance of Maximum Entropy parameters via typical error propagation techniques of $\sigma_{Y}^{2}=\sum_{i}{\left(\frac{\partial Y}{\partial J_{i}}\right)}^{2}\sigma_{J_{i}}^{2}$, where $J_{i}$ are Maximum Entropy parameters and *Y* is the performance metric. As such, $\sigma_{Y}^{2}$ asymptotically goes as 1/*N*, meaning that the variance in performance metrics goes as 1/*N*. To estimate Maximum Entropy parameters $J_{i}$, we need to populate means and covariances and potentially some higher-order moments, depending on the Maximum Entropy model, which means that we need to estimate a number of elements that increases as a power law in the dimensionality of the stimulus. Therefore, *N* needs only increase in power law fashion with the dimensionality of the stimulus to maintain accuracy.

In other words, the estimators introduced here are consistent and also have a variance that decays as 1/*N* and a bias that decays at most as 1/*N*, where *N* is the amount of data points. This alone does not provide us with a computational benefit over mutual information estimation. However, if we examine how *N* must change to maintain accuracy as the dimensionality of the data increases, we find an immense computational benefit. With mutual information estimates, *N* must increase exponentially with the dimensionality with many estimators. The reason for this is that histograms over bins that increase in an exponential fashion with the dimensionality of the environmental input and sensor state are estimated in order to understand mutual information. Meanwhile, for these biosensor metrics here, *N* need only increase in a power law fashion. For very high-dimensional datasets, confusion matrices have a computational advantage. The only caveat is that sometimes, Maximum Entropy estimation is difficult because the state space grows exponentially with the dimensionality of the environmental input and sensor state, but there are well-known workarounds for this such as contrastive divergence [29] that we did not use here.

## Discussion

There were two main justifications for using bias and variance or confusion matrices instead of mutual informations. First, bias and variance or confusion matrices provide more information than mutual information, replacing a single number and a hard-to-interpret capacity-achieving distribution in the case that channel capacity is computed with a suite of numbers that describe how precision and accuracy both change as the environmental input changes. Second, estimation of bias and variance or confusion matrices may actually be easier than estimation of notoriously difficult-to-estimate mutual informations.

It is worth elaborating upon that second point — the statistical advantages of this new method. When statistical mechanical models are used as stimulus-dependent Maximum Entropy models, estimation errors in the bias and variance or confusion matrices result from errors in the statistical mechanical models. These errors cannot be reasonably compared with error in estimating mutual informations, since the errors from statistical mechanical models come from errors in applying underlying physical principles and not estimation errors from limited data. However, in a more comparable case, when data-driven stimulus-dependent Maximum Entropy models are considered, estimating bias and variance or confusion matrices have a slight advantage, even in the limit that the number of data points *N* is large. Mutual information estimators have a bias and variance that decay as 1/*N*, but a curse of dimensionality causes maintaining accuracy in mutual information estimates to require an exponential growth in *N* as the dimensionality of the stimulus increases because the estimators used are implicitly estimating probability distributions or approximations thereof [6]. Some more favorable mutual information estimation procedures that operate on different principles may have different convergence properties based on aspects of the data such as the number of coincidences [30] or more severe requirements if the dimensionality of the problem grows with the amount of data [6]. On the other hand, our estimators may have similar variance scalings, but do not require exponentially large amounts of data as dimensionality of the stimulus increases. And so, the scalings of errors in bias and variance or confusion matrices are superior to those of mutual informations.

This new method compares favorably to mutual information estimation in terms of computational complexity. When statistical mechanical models are known *a priori*, parameter estimation reduces to a standard least-squares fit of binding or activity curves — sometimes requiring as few as ten data points, given the specificity of biophysically derived functional forms. For data-driven Maximum Entropy models, the primary bottleneck is partition function computation: in our neural network application, we computed the partition function using a greedy neuron selection procedure similar to Ref [17], whose computational requirements grow faster than exponentially in the number of candidate neurons. However, estimation of coupling constants in spin-glass Ising models is a well-studied problem with efficient solutions. Contrastive divergence [29] greatly eases computation of spin-glass weights, and Minimum Probability Flow [31] or Minimum Energy Flow [32] provide extremely compute-efficient alternatives to exact partition function calculation. Adoption of these methods would allow the framework to scale to much larger neural populations while retaining the interpretive advantages demonstrated here. Also, further improvements to Minimum Probability Flow [31] or Minimum Energy Flow [32] will allow for extremely compute-efficient alternatives to exact calculation of the partition function.

Although this new method introduces new quantities that may be hard to interpret, there are natural interpretations of bias and variance with biological significance in the context of biosensing. Bias reflects accuracy, and so if an environmental stimulus has low bias, it is estimated accurately; meanwhile, an environmental stimulus with high bias is estimated inaccurately. Variance reflects precision, and so if an environmental stimulus has low variance, it is estimated with high precision; meanwhile, an environmental stimulus with high variance is estimated with low precision. The bias, variance, and mean-squared error as a function of environmental stimulus reveals which environmental stimuli the biosensor is set up to estimate best. Therefore, for example, bacterial chemotactic receptors are excellent at estimating low concentrations of chemoattractant but find it difficult to estimate high concentrations of chemoattractant accurately even though they estimate these high concentrations with confidence. On the other hand, our analyses show that nACh receptors are excellent estimators of both low and high concentrations of neurotransmitter, but relatively bad estimators of middling concentrations of neurotransmitter. And genetic regulatory circuits are better and better estimators of higher and higher concentrations of the number of repressor molecules, which is a direct readout of how much sugar there is in the environment. Meanwhile, the confusion matrices reveal not only predictive accuracies, but more fine-grained metrics that explain how likely the network is to mistake stimulus silence for stimulus activity and vice versa.

The framework introduced here is broadly applicable beyond the specific biosensors analyzed. Any biological system whose response can be modeled as a conditional distribution p(s∣x), even if it maps input time series to output time series, is immediately amenable to this analysis. Other sensory systems that could be studied using these methods include: olfactory receptors, whose dose-response curves are well-described by MWC-type models [24] and for which bias and variance as a function of odorant concentration would clarify which odors are encoded reliably; photoreceptors, where the ordinal variable of light intensity and well-characterized phototransduction cascades make bias-variance analysis straightforward, e.g., using results of Ref [33]; and hair cells of the auditory and vestibular systems, where the ordinal structure of frequency tuning makes bias and variance analyses a natural fit [34]. Beyond individual receptors, the spin-glass MaxEnt framework applied here to cultured cortical neurons could in principle be extended to *in vivo* recordings, to other brain regions, or to other organisms entirely, provided that partition function computation is handled via contrastive divergence or related methods. More broadly, any engineered or evolved sensor system — including those in synthetic biology or neuromorphic computing — for which a generative model of sensor-stimulus relationships can be fit stands to benefit from this style of analysis.

## Conclusion

We applied and investigated a combination of stimulus-dependent Maximum Entropy models [12] or joint Maximum Entropy models with maximum likelihood estimation and the usual statistical metrics on both ordinal and categorical variables to understand how well sensors represent environmental information. These methods use the traditional Maximum Entropy models [11,12] to understand how a homunculus might see the environment to provide an information-rich alternative to mutual information assessments. This method reveals not only that sensors represent some amount of information about the environment, but also how much this amount of information varies and what type of information is provided based on the environmental input.

When the environmental input was ligand concentration, the analysis revealed how much bias and variance was present in the sensory estimator’s implicit understanding of the environment. We analyzed a number of MWC receptors as in Ref [24] and found new insights into exactly how well these receptors represented their ligand concentrations. In particular, we found that while a mutual information analysis shows that independent receptors without cooperativity transmit more information about the environmental input, the analyses here showed that some ligand concentrations were better estimated with cooperative receptors based on bias and variance metrics.

We looked at cultured neurons stimulated by a point process stimulus and analyzed its memory and predictive power with this new analysis as opposed to the mutual information analysis in Ref [17]. We treated the joint stimulus and sensor with a spin-glass Ising model to quantify memory and prediction via confusion matrices, with true positive, true negative, and predictive accuracies visualized as a function of time-shift, subsystem size, and stimulation type. These metrics yielded results consistent with post-stimulus electrode spiking distributions observed in Ref [17] but additionally quantified how strongly the network encodes memory of the stimulus through illustrating exact numerical changes in true positive and negative rates with time-shift. Moreover, increasing subsystem size reveals improvements in average predictive accuracy of the network, which has not been thoroughly investigated yet. Partitioning experiments into hourly intervals showed that networks represent the stimulus in a way that is stable over the entire experiment.

See Table 2 for a comparison of this new method with previous mutual information-based methods. Mutual information is notoriously difficult to estimate [6,35]. Compared to the mutual information approach [17], we faced no computational difficulties when calculating ordinal quantities with ligand-receptor binding, but did face computational difficulties in developing estimators for categorical variables. Computational difficulties primarily stemmed from calculating the partition function, whose computational complexity scales exponentially with system size. Estimating confusion matrix metrics using Minimum Probability Flow [31] or the closely-related Minimum Energy Flow [32] — a more compute-friendly parameter estimation method — was also tried, but ultimately was not fruitful because it failed to correctly predict the functional connectivity between neurons and stimulus, despite the results of Ref [10]. Contrastive divergence is a possible workaround to such problems [29].

**Table 2:** Comparison of mutual information, bias/variance/MSE, and confusion matrix approaches to biosensor analysis.

	Mutual Information	Bias/Variance	Confusion Matrix
**Variable type**	Any	Ordinal	Categorical
**Output**	Single scalar	Function of stimulus value	$n^{2}-n$ Off-diagonal elements
**What it reveals**	Total information shared	Accuracy & precision at each stimulus level	Error type at each stimulus category
**Computational scaling with data *N***	Exponential in stimulus dimensionality	Power law in stimulus dimensionality	Power law in stimulus dimensionality
**Requires *p*(*x*)?**	No	Only for MAP; MLE does not	Only for MAP; MLE does not
**Interpretability**	Abstract	Intuitive (bias = accuracy, variance = precision)	Intuitive (true positive/negative rates)
**Main limitation**	Undersampling bias; one number hides stimulus-specific structure	Requires parametric sensor model	Exponential partition function for high-dimensional 𝐬

Despite computational difficulties in estimating stimulus-dependent [12] or joint Maximum Entropy models, our methodology nonetheless provides invaluable insights into memory and prediction in these biological systems. Mutual information analyses produce only one number to characterize the sensor’s entire performance, while our analysis reveals detailed information about how well the sensor deals with each type of environmental stimulus. Perhaps future efforts could focus on remedying this problem with mutual information analyses via a calculation of *I*[*S*;*X* = *x*], but even this fails to understand the benefits of cooperativity in receptors, for instance. As it stands, our new method is computationally tractable and produces additional insight into biological systems that would be impossible to glean otherwise.

More broadly, this framework represents a general-purpose toolkit for quantifying sensory encoding in any biological system admitting a statistical mechanical or stimulus-dependent Maximum Entropy model. The examples analyzed here — genetic regulatory circuits, ligand-gated ion channels, chemotactic receptors, and cultured neural networks — span several orders of magnitude in biological complexity, suggesting that bias, variance, and confusion matrices may serve as a unifying language for comparing sensory performance across systems and organisms. Future work could extend this framework in several directions: applying it to *in vivo* neural recordings where naturalistic stimulus statistics are available and can replace the uniform prior; developing efficient parameter estimation via contrastive divergence or Minimum Probability Flow to enable analysis of larger neural populations; and combining bias-variance analyses with an understanding of which stimuli the sensor often sees to ask not just how well a sensor performs, but whether its performance is matched to the statistics of its natural environment [36]. The latter direction connects naturally to broader questions in theoretical biology about the degree to which biological sensors are adapted to their ecological niches — questions that the stimulus-resolved metrics introduced here are particularly well suited to answer, adding to information maximization analyses that assume a particular *p*(*x*) and derive the information-theoretically optimal p(s∣x).
