# Supplementary material for: Analyzing an organism’s sensors using Maximum Entropy (MaxEnt) models with bias, variance, and confusion matrices
Source: PLoS One. 2026 Aug 11;21(8):e0342165. doi: 10.1371/journal.pone.0342165 (PMC13460584; doi:10.1371/journal.pone.0342165)
Supplement: Supplementary file 1 [file pone.0342165.s001.pdf]

Review

Title: *Analyzing an organism's sensors using Maximum Entropy models with bias, variance, and confusion matrices.*

General comments

In this manuscript authors show how sensory systems interact with stimuli via Maximum Entropy models in combination with Maximum Likelihood Estimation replacing MI, with bias, variance and confusion matrix. Authors have worked on bridging the interdisciplinary gap of biophysics, statistical inference and information theory.

However following improvements are required for the acceptance of this paper for publication:

- Clearly showing how results are improved between these two techniques. When MI fails and the suggested methods succeed
- Mathematical link between MI and the suggested methods
- What assumptions are made on mathematical and statistical models and their validation?
- Clear interpretation of results in real world situations
- Important features of the data and its structure need clarity
- How results can be generalized?
- How priors are chosen, under what circumstances?

Suggestions

- More mathematical clarity is required
- Add numerical comparison of MI vs Bias, Variance and confusion matrix results
- Disagreement of results between the two strategies
- Adding effect sizes, confidence intervals, power analysis, etc. can improve clarity
- Cross validation confusion matrices can be useful
- Add full pipeline description, selection and training of the model, tuning of hyperparameters, etc. to make results reproduceable.
- Notations and symbols need to be more consistent

Final Comments

Claim is very strong but without quantification.

Recommendation

Major Revision
