# Supplementary material for: Analyzing an organism’s sensors using Maximum Entropy (MaxEnt) models with bias, variance, and confusion matrices
Source: PLoS One. 2026 Aug 11;21(8):e0342165. doi: 10.1371/journal.pone.0342165 (PMC13460584; doi:10.1371/journal.pone.0342165)
Supplement: Supplementary file 3 [file pone.0342165.s004.docx]

Dear Editors,

Thank you for the chance to revise. All our comments are in italics.

**Comments to the Author**

1. If the authors have adequately addressed your comments raised in a previous round of review and you feel that this manuscript is now acceptable for publication, you may indicate that here to bypass the “Comments to the Author” section, enter your conflict of interest statement in the “Confidential to Editor” section, and submit your "Accept" recommendation.

Reviewer #1: All comments have been addressed

Reviewer #4: (No Response)

2. Is the manuscript technically sound, and do the data support the conclusions?

The manuscript must describe a technically sound piece of scientific research with data that supports the conclusions. Experiments must have been conducted rigorously, with appropriate controls, replication, and sample sizes. The conclusions must be drawn appropriately based on the data presented.

Reviewer #1: Yes

Reviewer #4: (No Response)

3. Has the statistical analysis been performed appropriately and rigorously?

Reviewer #1: Yes

Reviewer #4: (No Response)

4. Have the authors made all data underlying the findings in their manuscript fully available?

The [PLOS Data policy](https://nam02.safelinks.protection.outlook.com/?url=http%3A%2F%2Ftrack.editorialmanager.com%2FCL0%2Fhttp%3A%252F%252Fwww.plosone.org%252Fstatic%252Fpolicies.action%2523sharing%2F1%2F010f019dce98218c-a761f763-df99-4807-a916-8e6f95433ca9-000000%2FQMYWzM-zgwE5kJPcYvEGPJhKnIXUVwxgNmcEids4rXQ%3D258&data=05%7C02%7Csmarzen%40natsci.claremont.edu%7Cc6a16cba96b246c238ad08dea44c0ec0%7C47274664281d4e3282489661a922b78c%7C0%7C0%7C639128844041841212%7CUnknown%7CTWFpbGZsb3d8eyJFbXB0eU1hcGkiOnRydWUsIlYiOiIwLjAuMDAwMCIsIlAiOiJXaW4zMiIsIkFOIjoiTWFpbCIsIldUIjoyfQ%3D%3D%7C0%7C%7C%7C&sdata=myfMk3cxQ%2BLYkHIdV4noGvJV8%2B8HGao9T69SQ8fTYtg%3D&reserved=0) requires authors to make all data underlying the findings described in their manuscript fully available without restriction, with rare exception (please refer to the Data Availability Statement in the manuscript PDF file). The data should be provided as part of the manuscript or its supporting information, or deposited to a public repository. For example, in addition to summary statistics, the data points behind means, medians and variance measures should be available. If there are restrictions on publicly sharing data—e.g. participant privacy or use of data from a third party—those must be specified.

Reviewer #1: Yes

Reviewer #4: (No Response)

5. Is the manuscript presented in an intelligible fashion and written in standard English?

PLOS ONE does not copyedit accepted manuscripts, so the language in submitted articles must be clear, correct, and unambiguous. Any typographical or grammatical errors should be corrected at revision, so please note any specific errors here.

Reviewer #1: Yes

Reviewer #4: (No Response)

6. Review Comments to the Author

Please use the space provided to explain your answers to the questions above. You may also include additional comments for the author, including concerns about dual publication, research ethics, or publication ethics. (Please upload your review as an attachment if it exceeds 20,000 characters)

Reviewer #1: The authors have thoroughly addressed all previous concerns. The revised manuscript is significantly improved.

*Thank you.*

Reviewer #4: Journal: PLOS ONE
MS ID: PONE-D-26-02593R1
Title: Analyzing an organism’s sensors using Maximum Entropy models with bias, variance,
and confusion matrices
Version: R1 Date: 21 April 2026

Reviewer's report:
Comment 1—The authors would need to cite any empirical benchmarks comparing the runtime of their method with that of MI estimators on the same data.

*Most of the asymptotic work is theoretical, so we have cited the appropriate papers now for a comparison in the large data limit. We reserve other limits that are of specialty interest such as when the number of parameters grows as the number of data points increases for future work, as it is beyond the scope of this paper to tackle that question as well. We wrote:*

*“\textcolor{blue}{Mutual information estimators have a bias and variance that decay as $1/N$, but a curse of dimensionality causes maintaining accuracy in mutual information estimates to require an exponential growth in $N$ as the dimensionality of the stimulus increases because the estimators used are implicitly estimating probability distributions or approximations thereof \cite{paninski2003estimation}. Some more favorable mutual information estimation procedures that operate on different principles may have different convergence properties based on aspects of the data such as the number of coincidences \cite{nemenman2011coincidences} or more severe requirements if the dimensionality of the problem grows with the amount of data \cite{paninski2003estimation}. On the other hand, our estimators may have similar variance scalings, but do not require exponentially large amounts of data as dimensionality of the stimulus increases. And so, the scalings of errors in bias and variance or confusion matrices are superior to those of mutual informations.}”*

Comment 2—Adding a brief convergence analysis would substantially strengthen the manuscript's credibility, particularly given the authors' emphasis on computational claims.

*We now do this in a new subsection:*

*“\subsection*{Convergence properties of new biosensor metrics}*

*\textcolor{blue}{Maximum Entropy parameters are in fact maximum likelihood estimators in that they best fit exponential linear models to data. Because maximum likelihood estimators are consistent, we find that the estimators here (functions of a maximum likelihood estimator) are consistent. Also, maximum likelihood estimators are asymptotically normal with variance that goes as $1/N$ and a bias that does at worst the same \cite{cramer1999mathematical}. Here we offer a sketch of a proof for how that translates to convergence of the estimators of bias, variance, mean-squared error, and confusion matrices. In the limit that the amount of data is large, $1/N$ is quite small, and we are justified in making a small noise approximation in which statistics are roughly Gaussian. In particular, confusion matrix elements or bias, variance, and mean-squared error can be said to vary linearly with the variance of Maximum Entropy parameters via typical error propagation techniques of $\sigma_{Y}^2 = \sum_i \left(\frac{\partial Y}{\partial J_i}\right)^2\sigma_{J_i}^2$, where $J_i$ are Maximum Entropy parameters and $Y$ is the performance metric. As such, $\sigma_Y^2$ asymptotically goes as $1/N$, meaning that the variance in performance metrics goes as $1/N$. To estimate Maximum Entropy parameters $J_i$, we need to populate means and covariances and potentially some higher-order moments, depending on the Maximum Entropy model, which means that we need to estimate a number of elements that increases as a power law in the dimensionality of the stimulus. Therefore, $N$ needs only increase in power law fashion with the dimensionality of the stimulus to maintain accuracy..}*
