# Supplementary material for: Analyzing an organism’s sensors using Maximum Entropy (MaxEnt) models with bias, variance, and confusion matrices
Source: PLoS One. 2026 Aug 11;21(8):e0342165. doi: 10.1371/journal.pone.0342165 (PMC13460584; doi:10.1371/journal.pone.0342165)
Supplement: Supplementary file 4 [file pone.0342165.s003.docx]

Dear Editor,

Attached is our response to reviewers. Responses are in italics. We hope we have answered all questions and that the article is worthy of review and hopefully publication. Also, our new Funding Statement is as follows:

This work was performed in part at the Aspen Center for Physics and was supported by a grant from the Alfred P. Sloan Foundation (G-2024-22395). Many thanks to the Pioneer Academy for support and hospitality. This study was supported by the US Air Force Office for Scientific Research, Grant Number FA9550-19-1-0411. Research was sponsored by the Army Research Office and was accomplished under Grant Number W911NF-25-1-0260. The views and conclusions contained in this document are those of the authors and should not be interpreted as representing the official policies, either expressed or implied, of the Army Research Office or the U.S. Government. The U.S. Government is authorized to reproduce and distribute reprints for Government purposes notwithstanding any copyright notation herein. The funders had no role in study design, data collection and analysis, decision to publish, or preparation of the manuscript.

Reviewer #1: Review of the Manuscript (PONE-D-26-02593) titled
Analyzing an organism's sensors using Maximum Entropy models with bias, variance, and confusion matrices

This manuscript proposes an alternative framework to mutual information (MI) for analyzing biological sensors, using stimulus-dependent Maximum Entropy (MaxEnt) models combined with maximum likelihood estimation to derive estimators of environmental states. These estimators are then evaluated using bias/variance metrics (for ordinal stimuli) or confusion matrices (for categorical stimuli). The authors apply this approach to ligand-receptor binding models and cultured neuronal networks. The manuscript in its current form suffers from significant methodological and statistical weaknesses that must be checked prior to publication.

*We have endeavored to fix these issues.*

• Insufficient statistical justification for replacing mutual information with bias/variance and confusion matrices, without adequately addressing their own estimation biases or undersampling issues.

*We have added to the Discussion:*

*\textcolor{blue}{There were two main justifications for using bias and variance or confusion matrices instead of mutual informations. First, bias and variance or confusion matrices provide more information than mutual information, replacing a single number and a hard-to-interpret capacity-achieving distribution in the case that channel capacity is computed with a suite of numbers that describe how precision and accuracy both change as the environmental input changes. Second, estimation of bias and variance or confusion matrices may actually be easier than estimation of notoriously difficult-to-estimate mutual informations.}*

*\textcolor{blue}{It is worth elaborating upon the statistical advantages of this new method. When statistical mechanical models are used as stimulus-dependent Maximum Entropy models, estimation errors in the bias and variance or confusion matrices result from errors in the statistical mechanical models. These errors cannot be reasonably compared with error in estimating mutual informations, since the errors from statistical mechanical models come from errors in applying underlying physical principles and not estimation errors from limited data. However, in a more comparable case, when data-driven stimulus-dependent Maximum Entropy models are considered, estimating bias and variance or confusion matrices have a slight advantage, even in the limit that the number of data points $N$ is large. The best mutual information estimators have a bias that decays as $1/N$, but a curse of dimensionality causes maintaining accuracy in mutual information estimates to require an exponential growth in $N$ as the dimensionality of the stimulus increases. On the other hand, covariance and mean estimators are typically unbiased and usually only require that $N$ increase as a power law with the dimensionality of the stimulus. Errors in estimating mean and covariance propagate linearly in the small-noise (Gaussian) approximation to errors in estimating Maximum Entropy weights, and thus errors in estimating bias and variance or confusion matrix elements, and so the scalings of errors in bias and variance or confusion matrices are superior to those of mutual informations.}*

• The method still faces exponential complexity in partition function calculation, limiting scalability, and relies on ad hoc neuron subset selection.

*We have now written in the Discussion:*

*\textcolor{blue}{This new method may also be advantageous in terms of compute efficiency if state-of-the-art algorithms are employed. Statistical mechanical models, if they have energies or dissociation constants that require estimation, can typically be completed with least-squares estimates that fit binding or activity curves to data. Sometimes, these estimates can be quite accurate even with only ten data points because the functional form for binding curves or activity curves dictated by biophysics is so specific. As for the compute-intensive spin-glass Ising models that can be used as stimulus-dependent Maximum Entropy models of neural activity, there may be a compute benefit to using our new method if better parameter estimation methods are employed. We happened to calculate the partition function in a method similar to that of Ref. \cite{lamberti2023prediction}, which required greedily choosing neurons that most increased the predictive accuracy. This method has exponential compute requirements in the number of neurons that you consider adding. However, one can use contrastive divergence \cite{carreira2005contrastive} to greatly ease computation of weights in spin-glass Ising models. Also, further improvements to Minimum Probability Flow \cite{sohldickstein} or Minimum Energy Flow \cite{hillarhopfield} will allow for extremely compute-efficient alternatives to exact calculation of the partition function.}*

• Methodologically weak subset selection based only on Pearson correlation, which fails to capture multivariate or dynamic predictive relationships and likely explains discrepancies with prior predictive findings.

*We have now fixed this in the Results by utilizing a method similar to that of the PNAS Nexus that used mutual informations. Now, we greedily add neurons that most increase the predictive accuracy*. *We say this:*

*\textcolor{blue}{To choose a subset, we greedily add neurons based on how much they contribute to improving predictive accuracy of our estimator, similar to the algorithm used in Ref. \cite{lamberti2023prediction}. Starting with just the stimulus neuron, we first exhaustively iterate over a subset of two neurons from the pool of 59 neurons, choosing the neuron that best improves predictive accuracy. This neuron is appended to the subset, fixed in place, and an exhaustive search is now done with a three-neuron subset by examining each of the remaining 58 options. This process is repeated until the desired subset size is obtained. With this method, subsystem sizes were chosen from three to six neurons.} The MaxEnt model was then fitted to the data, and the confusion matrix \textcolor{blue}{elements} characterizing the estimator's performance are shown in Fig \ref{fig:cm metrics}.*

• No correction for multiple comparisons, incomplete handling of class imbalance in confusion matrices, and insufficient comparison with mutual information benchmarks.

*We have now used the Bonferroni correction for the multiple comparisons that we did in first comparing controls with the optical and then electrical with the optical, replacing the p of 0.05 with a p of 0.025. We have added in Results:*

*Statistics of model metrics over experiments were explored to compare optogenetic and electrical trials. Only two of the time-shifts in Fig \ref{fig:cm metrics}, $-100$ and $0$ ms, are relevant: optogenetic and electrical predictive accuracies are not at all distinguishable at other time-shifts. At a time-shift of 0 ms and a subsystem size of six neurons, \textcolor{blue}{the model for electrical stimulation was normally distributed. With $\alpha=0.05$, performing a Shapiro-Wilk normality test on electrically stimulated samples yielded a $p$-value of $p=0.0998$. However, the same test on optogenetic samples yielded a $p$-value of $p=1.13\cdot 10^{-7}$, indicating non-normality. Since the optogenetic stimulation samples are not normally distributed, to compare the location parameters of electrical and optogenetic stimulation, we first check that each distribution is distinct via the Mann-Whitney $U$ test, which is confirmed with a $p$-value of $p=6.16\cdot 10^{-4}$. Using the Hodges-Lehmann estimator, we then find a $1.71\%$ higher predictive accuracy in electrically stimulated samples compared to optogenetically stimulated samples. This is statistically significant even with a Bonferroni correction for multiple comparisons so that the correct significance level is $p=0.025$. Considering baseline accuracy already lies at about $97.85\%$, this improvement is equivalent to a fivefold decrease in error rates. We can interpret this as follows: a locally concentrated electrical pulse interacts strongly with a subset of the neurons present, while light pulses interact weakly with the whole system; as such, it is significantly easier to predict behavior in electrical stimulation.} Meanwhile, there was no statistically significant difference between globally stimulated cultures and control trials (\textcolor{blue}{$p=0.319$ using the Mann-Whitney $U$ test}).*

*\textcolor{blue}{Repeating this statistical analysis at $-100$ ms and again at six neurons, model predictive accuracy outputs were normally distributed for optogenetic stimulation but not for electrical stimulation: Shapiro-Wilk tests revealed $p=1.26\cdot 10^{-4}$ and $p=0.0627$ for electrical and optogenetic stimulation, respectively. The Mann-Whitney $U$ test applied on the optogenetic and electrical model samples indicates similar distributions with $p=0.122$, but optogenetic samples show a statistically significant difference from baseline with $p=0.0214$ using Welch's $t$-test. Interestingly, electrical model samples are almost identical to baseline, with a Mann-Whitney $U$ test $p$ value of $p=1$.}*

*Class imbalance is now handled:*

*\textcolor{blue}{To account for class imbalance (the stimulus is much more likely to be inactive than active), we weighted the KL divergence appropriately such that inaccuracies in guessing activity were penalized proportionately. Moreover, we used the \texttt{scipy.minimize} module for model fitting with a custom-designed \texttt{KL} function to account for class imbalance. In the minimization process, we used the \texttt{L-BFGS-B} optimizer method and ran a maximum of 200 iterations or until the difference between successive iterations was less than a set tolerance of $10^{-7}$. Specifics of implementation can be found in Supplementary Information S1.}*

• Inadequate treatment of temporal dependence due to binning neural activity into independent windows, ignoring autocorrelation that could violate model assumptions.

*This method does not actually ignore autocorrelations, although it seems to be not including multiple time bins in the model. The question is whether or not the model captures the dependencies between the stimulus now and the network activity previously or in the future. The method automatically does that by having covariance matrices that capture the correlations that relate the stimulus now to the network activity in the future or the past. If there are autocorrelations in the network activity, these will automatically affect the covariance matrices for the present stimulus and the network activity in the future. The PNAS Nexus mutual information estimates have a similar underlying logic, as mutual informations were estimated for the stimulus now and the network activity in the future or the past without building a model of the network activity’s autocorrelation by simply calculating mutual informations between the stimulus now and the network activity in the future or the past. Both the PNAS Nexus results and the results here therefore automatically account for these autocorrelations. If one desires to think of neural codewords as being spread over many time bins, then one can include many time bins in the stimulus-dependent Maximum Entropy model, but we did not do that here. That would be an interesting subject of future inquiry. We now write:*

*\textcolor{blue}{Time-shifting implicitly takes into account temporal autocorrelations in the network state and the stimulus state, as described in Ref. \cite{lamberti2023prediction}. If there are temporal autocorrelations in the network activity, they will automatically allow for potentially reduced correlations between past or future stimulus activity and present network activity. One way to think about this is to notice that we are essentially marginalizing a probability distribution over successive network states and successive stimulus states in order to retain just the probability distribution over past or future stimulus states and the present network state; but this can be just as well accomplished simply by fitting a Maximum Entropy model to past or future stimulus states and present network states without first fitting to successive network states and then marginalizing.}*

• Superficial biological interpretation of results without clear mechanistic or statistical explanations linking model outputs to biological function.

*The main idea is that bias reflects the accuracy with which an environmental stimulus can be represented and variance represents the precision with which an environmental stimulus can be represented. This is now described in more detail in the Discussion section:*

*\textcolor{blue}{Although this new method introduces new quantities that may be hard to interpret, there are natural interpretations of bias and variance with biological significance in the context of biosensing. Bias reflects accuracy, and so if an environmental stimulus has low bias, it is estimated accurately; meanwhile, an environmental stimulus with high bias is estimated inaccurately. Variance reflects precision, and so if an environmental stimulus has low variance, it is estimated with high precision; meanwhile, an environmental stimulus with high variance is estimated with low precision. The bias, variance, and mean-squared error as a function of environmental stimulus reveals which environmental stimuli the biosensor is set up to estimate best. Therefore, for example, bacterial chemotactic receptors are excellent at estimating low concentrations of chemoattractant but find it difficult to estimate high concentrations of chemoattractant accurately even though they estimate these high concentrations with confidence. On the other hand, our analyses show that nACh receptors are excellent estimators of both low and high concentrations of neurotransmitter, but relatively bad estimators of middling concentrations of neurotransmitter. And genetic regulatory circuits are better and better estimators of higher and higher concentrations of the number of repressor molecules, which is a direct readout of how much sugar there is in the environment. Meanwhile, the confusion matrices reveal not only predictive accuracies, but more fine-grained metrics that explain how likely the network is to mistake stimulus silence for stimulus activity and vice versa.}*

Reviewer #2: A little observation about the confidence levels used : statisticians would prefer a 95% or 99% Confidence Interval to 90% confidence interval, as used in the analyses. I would suggest, since a significance level of 5% was used for the Shapiro-Wilk test and for consistency, that 95% confidence intervals be constructed instead of the 90%.

*Thank you! We have now done this.*

If possible Author(s) should compare their new method with earlier (existing) methods with empirical results before concluding their new method is superior.

*We have now added mutual information analyses of all ligand-receptor binding situations; see all additional Figures. Because data was not available for these situations on the ligand concentrations likely to be seen by the receptor, we did a channel capacity analysis, where we found the maximum mutual information possible given the channel and reported both the channel capacity itself and the capacity-achieving distribution. This capacity-achieving distribution is a little tricky to interpret, but one can roughly explain that perhaps the receptor “likes” the concentrations that are high in probability for the capacity-achieving distribution. Comparison of these capacity-achieving distributions with the mean-squared errors obtained from our new analysis are now in the main text. We have added these figures and numbers to the Results with this preamble:*

*\textcolor{blue}{We are unable in most of the cases to report mutual informations because data is not available on ligand concentration profiles that are likely to be seen by the receptors. However, given the receptor's biophysics as specified by the statistical mechanical model, there is a maximum mutual information that can be attained if the probability distribution over ligand concentrations is of a certain form. This maximum mutual information is the channel capacity, and the probability distribution that achieves this channel capacity is the capacity-achieving distribution. Thus, to compare with mutual information analyses, we calculate channel capacity and report both the channel capacity and the capacity-achieving distribution. Roughly speaking, one might say that receptors ``like'' the ligand concentrations for which the capacity-achieving probability is large, although this statement is unsubstantiated and hand-wavy at best. We therefore can compare the capacity-achieving distribution to the mean-squared error curves in order to understand the subtle differences between a mutual information analysis and our new analyses. Mean-squared error will be small if our analysis reveals that a particular ligand concentration is ``liked'' by the receptor, while the capacity-achieving distribution will be high if a mutual information analysis reveals that a certain ligand concentration is preferred. Standard code is available for calculating the capacity-achieving distribution and the channel capacity via the Blahut-Arimoto algorithm \cite{blahut1972computation} in the GitHub repository.}*

*The mutual information analysis of the neural data is contained in a previous publication which we referenced.*

*We have also included an additional paragraph in the Results section comparing and contrasting MaxEnt and Mutual Information:*

*\textcolor{blue}{Although we see nonzero predictive mutual information ($\text{MI}_\text{future}$) in Fig 3 of Ref \cite{lamberti2023prediction}, the mutual information relating to predictive capability is more than an order of magnitude smaller than the mutual information relating to memory capability ($\text{MI}_\text{future}$ compared to $\text{MI}_\text{past}$), with the largest $\text{MI}_\text{future}$ on the order of $10^{-3}$ and the largest $\text{MI}_\text{past}$ on the order of $10^{-1}$. The comparatively small magnitude of $\text{MI}_\text{future}$ means that the signal may never be strong enough to trigger the threshold of activity in the likelihood estimator of our MaxEnt model, resulting in model performance equivalent to baseline in the regime of positive time-shift. Meanwhile, there is enough mutual information encoded in $\text{MI}_\text{past}$ to trigger improvements above baseline, which is indicated by improved predictive accuracy in our model. The confusion matrix teases out more details with the addition of both true positive rates and true negative rates than the mutual information, indicating how often the network mistakes activity for silence and vice versa.}*

Reviewer #3: Suggestions
• More mathematical clarity is required

*We would appreciate some more feedback on what was unclear, please.*

• Add numerical comparison of MI vs Bias, Variance and confusion matrix results

*Please see all new figures and descriptions of these new results in the main text.*

• Disagreement of results between the two strategies

*Please see all new figures and descriptions of these new results in the main text.*

• Adding effect sizes, confidence intervals, power analysis, etc. can improve clarity

*These statistical issues are not relevant for statistical mechanical models, because those models are based not on data but on biophysical principles, but these concerns are relevant for the neural recordings. We now report 95% confidence intervals.*

• Cross validation confusion matrices can be useful
• Add full pipeline description, selection and training of the model, tuning of hyperparameters, etc. to make results reproduceable.

*We have now described how class imbalance was dealt with and specified relevant parameters for reproducibility. We now say this explicitly in Methods:*

*\textcolor{blue}{To account for class imbalance (the stimulus is much more likely to be inactive than active), we weighted the KL divergence appropriately such that inaccuracies in guessing activity were penalized proportionately. Moreover, we used the \texttt{scipy.minimize} module for model fitting with a custom-designed \texttt{KL} function to account for class imbalance. In the minimization process, we used the \texttt{L-BFGS-B} optimizer method and ran a maximum of 200 iterations or until the difference between successive iterations was less than a set tolerance of $10^{-7}$. Specifics of implementation can be found in Supplementary Information S1.}*

• Notations and symbols need to be more consistent

*Additional clarity on this comment would be so great, please, thank you.*

Final Comments
Claim is very strong but without quantification.

*Please see the Discussion. We have tried to make the evidence for the claim as clear as possible. Text below:*

*\section*{Discussion}*

*\textcolor{blue}{There were two main justifications for using bias and variance or confusion matrices instead of mutual informations. First, bias and variance or confusion matrices provide more information than mutual information, replacing a single number and a hard-to-interpret capacity-achieving distribution in the case that channel capacity is computed with a suite of numbers that describe how precision and accuracy both change as the environmental input changes. Second, estimation of bias and variance or confusion matrices may actually be easier than estimation of notoriously difficult-to-estimate mutual informations.}*

*\textcolor{blue}{It is worth elaborating upon that second point-- the statistical advantages of this new method. When statistical mechanical models are used as stimulus-dependent Maximum Entropy models, estimation errors in the bias and variance or confusion matrices result from errors in the statistical mechanical models. These errors cannot be reasonably compared with error in estimating mutual informations, since the errors from statistical mechanical models come from errors in applying underlying physical principles and not estimation errors from limited data. However, in a more comparable case, when data-driven stimulus-dependent Maximum Entropy models are considered, estimating bias and variance or confusion matrices have a slight advantage, even in the limit that the number of data points $N$ is large. The best mutual information estimators have a bias that decays as $1/N$, but a curse of dimensionality causes maintaining accuracy in mutual information estimates to require an exponential growth in $N$ as the dimensionality of the stimulus increases. On the other hand, covariance and mean estimators are typically unbiased and usually only require that $N$ increase as a power law with the dimensionality of the stimulus. Errors in estimating mean and covariance propagate linearly in the small-noise (Gaussian) approximation to errors in estimating Maximum Entropy weights, and thus errors in estimating bias and variance or confusion matrix elements, and so the scalings of errors in bias and variance or confusion matrices are superior to those of mutual informations.}*

*\textcolor{blue}{This new method may also be advantageous in terms of compute efficiency if state-of-the-art algorithms are employed. Statistical mechanical models, if they have energies or dissociation constants that require estimation, can typically be completed with least-squares estimates that fit binding or activity curves to data. Sometimes, these estimates can be quite accurate even with only ten data points because the functional form for binding curves or activity curves dictated by biophysics is so specific. As for the compute-intensive spin-glass Ising models that can be used as stimulus-dependent Maximum Entropy models of neural activity, there may be a compute benefit to using our new method if better parameter estimation methods are employed. We happened to calculate the partition function in a method similar to that of Ref. \cite{lamberti2023prediction}, which required greedily choosing neurons that most increased the predictive accuracy. This method has greater than exponential compute requirements in the number of neurons that you consider adding. However, one can use contrastive divergence \cite{carreira2005contrastive} to greatly ease computation of weights in spin-glass Ising models. Also, further improvements to Minimum Probability Flow \cite{sohldickstein} or Minimum Energy Flow \cite{hillarhopfield} will allow for extremely compute-efficient alternatives to exact calculation of the partition function.}*

*\textcolor{blue}{Although this new method introduces new quantities that may be hard to interpret, there are natural interpretations of bias and variance with biological significance in the context of biosensing. Bias reflects accuracy, and so if an environmental stimulus has low bias, it is estimated accurately; meanwhile, an environmental stimulus with high bias is estimated inaccurately. Variance reflects precision, and so if an environmental stimulus has low variance, it is estimated with high precision; meanwhile, an environmental stimulus with high variance is estimated with low precision. The bias, variance, and mean-squared error as a function of environmental stimulus reveals which environmental stimuli the biosensor is set up to estimate best. Therefore, for example, bacterial chemotactic receptors are excellent at estimating low concentrations of chemoattractant but find it difficult to estimate high concentrations of chemoattractant accurately even though they estimate these high concentrations with confidence. On the other hand, our analyses show that nACh receptors are excellent estimators of both low and high concentrations of neurotransmitter, but relatively bad estimators of middling concentrations of neurotransmitter. And genetic regulatory circuits are better and better estimators of higher and higher concentrations of the number of repressor molecules, which is a direct readout of how much sugar there is in the environment. Meanwhile, the confusion matrices reveal not only predictive accuracies, but more fine-grained metrics that explain how likely the network is to mistake stimulus silence for stimulus activity and vice versa.}*

Reviewer #4: Journal: PLOS ONE
MS ID: PONE-D-26-02593
Title: Analyzing an organism’s sensors using Maximum Entropy models with bias, variance,
and confusion matrices
Version: 1 Date: 16 February 2026

Reviewer's report:
The manuscript proposes an alternative framework to mutual information for analyzing the performance of biological sensors. The authors argue that while mutual information provides a single, often computationally intractable summary statistic, their method—which combines stimulus-dependent Maximum Entropy (MaxEnt) models with maximum likelihood estimation—offers a more detailed and computationally efficient picture. However, the manuscript has several issues that must be addressed before it can be considered for publication.

*Thank you. We hope that these changes are enough to warrant acceptance.*

Comment 1—The authors' claim of their method being “computationally efficient” is contradicted by their own text, indicating that the assertion of computational tractability is overstated. They have an obligation to demonstrate and quantify that efficiency. Currently, they do not. They state that mutual information is “computationally intractable” (Abstract) and “notoriously difficult to estimate” (Conclusion). They later admit to facing significant “computational difficulties” when applying their MaxEnt model (Conclusion). They even note that calculating the partition function “scales exponentially with system size”. The paper presents a paradox that a scalability study would resolve.

*We have now written the following asymptotic analyses in the new Discussion section:*

*\textcolor{blue}{There were two main justifications for using bias and variance or confusion matrices instead of mutual informations. First, bias and variance or confusion matrices provide more information than mutual information, replacing a single number and a hard-to-interpret capacity-achieving distribution in the case that channel capacity is computed with a suite of numbers that describe how precision and accuracy both change as the environmental input changes. Second, estimation of bias and variance or confusion matrices may actually be easier than estimation of notoriously difficult-to-estimate mutual informations.}*

*\textcolor{blue}{It is worth elaborating upon that second point-- the statistical advantages of this new method. When statistical mechanical models are used as stimulus-dependent Maximum Entropy models, estimation errors in the bias and variance or confusion matrices result from errors in the statistical mechanical models. These errors cannot be reasonably compared with error in estimating mutual informations, since the errors from statistical mechanical models come from errors in applying underlying physical principles and not estimation errors from limited data. However, in a more comparable case, when data-driven stimulus-dependent Maximum Entropy models are considered, estimating bias and variance or confusion matrices have a slight advantage, even in the limit that the number of data points $N$ is large. The best mutual information estimators have a bias that decays as $1/N$, but a curse of dimensionality causes maintaining accuracy in mutual information estimates to require an exponential growth in $N$ as the dimensionality of the stimulus increases. On the other hand, covariance and mean estimators are typically unbiased and usually only require that $N$ increase as a power law with the dimensionality of the stimulus. Errors in estimating mean and covariance propagate linearly in the small-noise (Gaussian) approximation to errors in estimating Maximum Entropy weights, and thus errors in estimating bias and variance or confusion matrix elements, and so the scalings of errors in bias and variance or confusion matrices are superior to those of mutual informations.}*

*\textcolor{blue}{This new method may also be advantageous in terms of compute efficiency if state-of-the-art algorithms are employed. Statistical mechanical models, if they have energies or dissociation constants that require estimation, can typically be completed with least-squares estimates that fit binding or activity curves to data. Sometimes, these estimates can be quite accurate even with only ten data points because the functional form for binding curves or activity curves dictated by biophysics is so specific. As for the compute-intensive spin-glass Ising models that can be used as stimulus-dependent Maximum Entropy models of neural activity, there may be a compute benefit to using our new method if better parameter estimation methods are employed. We happened to calculate the partition function in a method similar to that of Ref. \cite{lamberti2023prediction}, which required greedily choosing neurons that most increased the predictive accuracy. This method has greater than exponential compute requirements in the number of neurons that you consider adding. However, one can use contrastive divergence \cite{carreira2005contrastive} to greatly ease computation of weights in spin-glass Ising models. Also, further improvements to Minimum Probability Flow \cite{sohldickstein} or Minimum Energy Flow \cite{hillarhopfield} will allow for extremely compute-efficient alternatives to exact calculation of the partition function.}*

Comment 2—The authors state: “On the condition of normality, t-tests were used to determine the significance of differences”. The phrase “t-tests were used” is insufficient because there are several varieties of t-tests, each answering a different question and having different assumptions. Even if they specify “independent samples,” there is a second layer of ambiguity: Which version of the independent t-test (Student's/Welch's t-test)?

*Upon further analysis in accordance to other comments in the review, we have modified our statistical testing to account for non-normality, using the Mann-Whitney U test and Hodges-Lehmann estimator for location parameter differences. The t-test is now only used once, and we use Welch’s t-test for versatility.*

Comment 3—p is used twice in Equation (7), which is slightly confusing. While clear in context, a different symbol (e.g., p0) might improve readability.

*We have now changed $p$ to $p_0$.*

Comment 4—Equation 11 holds mathematically. However, if the model is simply fitting the joint distribution of two variables without explicitly handling the time dimension in the probability structure (i.e., treating them as simultaneous events), then using this joint probability for a time-shifted argmax might be statistically valid but biologically misaligned.

*This method does not actually ignore autocorrelations, although it seems to be not including multiple time bins in the model. The question is whether or not the model captures the dependencies between the stimulus now and the network activity previously or in the future. The method automatically does that by having covariance matrices that capture the correlations that relate the stimulus now to the network activity in the future or the past. If there are autocorrelations in the network activity, these will automatically affect the covariance matrices for the present stimulus and the network activity in the future. The PNAS Nexus mutual information estimates have a similar underlying logic, as mutual informations were estimated for the stimulus now and the network activity in the future or the past without building a model of the network activity’s autocorrelation by simply calculating mutual informations between the stimulus now and the network activity in the future or the past. Both the PNAS Nexus results and the results here therefore automatically account for these autocorrelations. If one desires to think of neural codewords as being spread over many time bins, then one can include many time bins in the stimulus-dependent Maximum Entropy model, but we did not do that here. That would be an interesting subject of future inquiry. We now write:*

*\textcolor{blue}{Time-shifting implicitly takes into account temporal autocorrelations in the network state and the stimulus state, as described in Ref. \cite{lamberti2023prediction}. If there are temporal autocorrelations in the network activity, they will automatically allow for potentially reduced correlations between past or future stimulus activity and present network activity. One way to think about this is to notice that we are essentially marginalizing a probability distribution over successive network states and successive stimulus states in order to retain just the probability distribution over past or future stimulus states and the present network state; but this can be just as well accomplished simply by fitting a Maximum Entropy model to past or future stimulus states and present network states without first fitting to successive network states and then marginalizing.}*

Comment 5—The paper should provide a direct, side-by-side comparison of their method to the MI on the same datasets. It would be straightforward to calculate the mutual information and compare it to the bias/variance plots. This would powerfully illustrate what information is "lost" or "gained" by using the more detailed metrics. How do the confusion matrix metrics relate to the MI values? Does a high true positive rate correlate with high MI? Such a comparison would ground the new method and demonstrate its added value concretely.

*Please see all new Figures and additional comments in the Results section.*

Comment 6—Pearson correlation assumes linearity and normality. Based on the manuscript's description of the data and the stated goals, is the application of Pearson correlation suitable or problematic?

*We have now changed the Pearson correlation method to one that is as similar as possible to the mutual information analysis to which the neural activity analysis should be compared. We now greedily add neurons that most increase the predictive accuracy. We write:*

*To develop an estimator, we used the Maximum Entropy spin-glass Ising model. Because this model requires calculation of the partition function, we chose a subset of the 60 neurons to construct the network vector $s$. Calculating the full partition function for $n$ neurons requires summing over all $2^{n}$ configurations of the system and, for our case of 60 neurons, is not computationally feasible. Therefore, it is worth investigating how much information can be captured with a subset of the entire population. \textcolor{blue}{To choose a subset, we greedily add neurons based on how much they contribute to improving predictive accuracy of our estimator, similar to the algorithm used in Ref. \cite{lamberti2023prediction}. Starting with just the stimulus neuron, we first exhaustively iterate over a subset of two neurons from the pool of 59 neurons, choosing the neuron that best improves predictive accuracy. This neuron is appended to the subset, fixed in place, and an exhaustive search is now done with a three-neuron subset by examining each of the remaining 58 options. This process is repeated until the desired subset size is obtained. With this method, subsystem sizes were chosen from three to six neurons.} The MaxEnt model was then fitted to the data, and the confusion matrix \textcolor{blue}{elements} characterizing the estimator's performance are shown in Fig \ref{fig:cm metrics}.*

Comment 7—The statement about a 1.31% improvement in predictive accuracy. While statistically significant, is this improvement biologically or practically meaningful, given that both are near 99%? A brief comment on effect size would be useful.

*We have now addressed the effect size in the Results section by appending the following:*

*\textcolor{blue}{...*

*Considering baseline accuracy already lies at about $97.85\%$, this improvement is equivalent to a fivefold decrease in error rates. We can interpret this as follows: a locally concentrated electrical pulse interacts strongly with a subset of the neurons present, while light pulses interact weakly with the whole system; as such, it is significantly easier to predict behavior in electrical stimulation.}*
