# Supplementary material for: Analyzing an organism’s sensors using Maximum Entropy (MaxEnt) models with bias, variance, and confusion matrices
Source: PLoS One. 2026 Aug 11;21(8):e0342165. doi: 10.1371/journal.pone.0342165 (PMC13460584; doi:10.1371/journal.pone.0342165)
Supplement: Supplementary file 6 [file pone.0342165.s006.docx]

Dear Editor,

Thank you for another round of review. We have responded to all queries from the reviewer, as well as sending our paper to a colleague and clarifying additional details based on his comments. We hope the paper is finally worthy of acceptance!

Our comments are in italics and reviewer comments are in regular font below.

Sincerely,

Chris Wang and Sarah Marzen

Reviewer #5: 1- The manuscript is scientifically sound, and the proposed methodology is novel and relevant to computational biology and statistical physics.

*Thank you so much. We tried to use your additional comments to improve the manuscript.*

2- The justification for replacing reciprocal information with bias, variance, and confusion matrices is compelling; nonetheless, the merits and drawbacks of the proposed framework may be summarised more explicitly in the Discussion or Conclusion.

*See Table 2 in the Conclusions now that summarizes the benefits and drawbacks of the two methods, reproduced here:*

*\begin{table}[h]*

*\centering*

*\caption{\textcolor{blue}{Comparison of mutual information, bias/variance/MSE, and confusion matrix approaches to biosensor analysis.}}*

*\label{tab:comparison}*

*\begin{tabular}{p{2.9cm}p{2.9cm}p{2.9cm}p{2.9cm}}*

*& \textbf{Mutual Information} & \textbf{Bias/Variance} & \textbf{Confusion Matrix} \\*

*\textbf{Variable type}*

*& Any*

*& Ordinal*

*& Categorical \\[6pt]*

*\textbf{Output}*

*& Single scalar*

*& Function of stimulus value*

*& $n^2 - n$ off-diagonal elements \\[6pt]*

*\textbf{What it reveals}*

*& Total information shared*

*& Accuracy \& precision at each stimulus level*

*& Error type at each stimulus category \\[6pt]*

*\textbf{Computational scaling with data $N$}*

*& Exponential in stimulus dimensionality*

*& Power law in stimulus dimensionality*

*& Power law in stimulus dimensionality \\[6pt]*

*\textbf{Requires $p(x)$?}*

*& No*

*& Only for MAP; MLE does not*

*& Only for MAP; MLE does not \\[6pt]*

*\textbf{Interpretability}*

*& Abstract*

*& Intuitive (bias $=$ accuracy, variance $=$ precision)*

*& Intuitive (true positive/negative rates) \\[6pt]*

*\textbf{Main limitation}*

*& Undersampling bias; one number hides stimulus-specific structure*

*& Requires parametric sensor model*

*& Exponential partition function for high-dimensional $\mathbf{s}$ \\*

*\end{tabular}*

*\end{table}*

3- The suggested method's computational complexity for large-scale biological networks should be briefly discussed by the authors, along with its practical scalability.

*Added:*

*\textcolor{blue}{This new method compares favorably to mutual information estimation in terms of computational complexity. When statistical mechanical models are known \textit{a priori}, parameter estimation reduces to a standard least-squares fit of binding or activity curves — sometimes requiring as few as ten data points, given the specificity of biophysically-derived functional forms. For data-driven Maximum Entropy models, the primary bottleneck is partition function computation: In our neural network application, we computed the partition function using a greedy neuron selection procedure similar to Ref.~\cite{lamberti2023prediction}, whose computational requirements grow faster than exponentially in the number of candidate neurons. However, estimation of coupling constants in spin-glass Ising models is a well-studied problem with efficient solutions. Contrastive divergence \cite{carreira2005contrastive} greatly eases computation of spin-glass weights, and Minimum Probability Flow \cite{sohldickstein} or Minimum Energy Flow \cite{hillarhopfield} provide extremely compute-efficient alternatives to exact partition function calculation. Adoption of these methods would allow the framework to scale to much larger neural populations while retaining the interpretive advantages demonstrated here.} Also, further improvements to Minimum Probability Flow \cite{sohldickstein} or Minimum Energy Flow \cite{hillarhopfield} will allow for extremely compute-efficient alternatives to exact calculation of the partition function.*

4-Some mathematical symbols and notation should be defined immediately when first introduced to improve readability for non-specialist readers.

*Thank you for the clarification request. We have gone back and added the following to best clarify possibly confusing symbols and notation:*

*1.*

*\begin{equation}\textcolor{blue}{*

*p_{\mathrm{model}}(x, \vb{s}) = \frac{1}{Z} \exp\left(-E_{\theta}(x, \vb{s})\right)}*

*\end{equation}*

*\textcolor{blue}{where $Z$ is the partition function, or the sum of $\exp(-E_\theta(x,\vb{s}))$ over all values of $x$ and $\vb{s}$, which serves as a normalization factor. Assuming the distribution is constrained and parametrized by a set of parameters $\theta$, we aim to} fit the data distribution…*

*2.*

*\textcolor{blue}{Here, $J$ is decomposed into four block matrices. Assuming a system size of $n$, $J_{xx}$ is a scalar interpreted as the stimulus firing propensity, $J_{sx} = J_{xs}^\top$ are $n-1$-dimensional vectors that represent stimulus-network couplings, and $J_{ss}$ is an $n-1\times n-1$ matrix describing the couplings between network elements.}*

*3.*

*The output of the statistical mechanical model \cite{garcia2011quantitative} is the following model for $p_\text{model}(s \mid x)$, where $s$ is whether or not RNAP is bound and $x$ is the number of \textcolor{blue}{Lac repressor (lacR) tetramers:*

*\begin{equation}*

*p_\text{model}(s \mid x) = \frac{p_0}{1+p_0+\frac{x}{N_\text{NS}}\exp(-\beta\Delta\epsilon_\text{rd})}.*

*\end{equation}*

*In the case of a single sensor, we may switch from the boldfaced $\vb{s}$ to $s$ to denote sensor state. Here, $p_0$ is the number of RNAP molecules, $P$, divided by the number of nonspecific DNA sites, $N_\mathrm{NS}$, with values of $p_0\approx 10^{-3}$ and $N_\text{NS}\approx 5\times 10^6$, and $\Delta\epsilon_\text{rd}$ is the repressor-DNA binding energy difference, which adopts a range of values that can be tuned in experiments \cite{garcia2011quantitative}. Finally, $\beta=1/k_B T$, the inverse temperature.}*

*4.*

*\textcolor{blue}{To briefly contextualize: $x$ is the ligand concentration, $K$ is the dissociation constant, $\Delta \epsilon$ is the cooperativity energy, and $\beta = 1/k_B T$.}*

5- In the Discussion, the biological implications of the results might be further highlighted, especially with regard to possible applications to other sensory systems outside of the examples given.

*Added:*

*\textcolor{blue}{The framework introduced here is broadly applicable beyond the specific biosensors analyzed. Any biological system whose response can be modeled as a conditional distribution $p(s∣x)$, even if it maps input time series to output time series, is immediately amenable to this analysis. Other sensory systems that could be studied using these methods include: olfactory receptors, whose dose-response curves are well-described by MWC-type models \cite{martins2011trade} and for which bias and variance as a function of odorant concentration would clarify which odors are encoded reliably; photoreceptors, where the ordinal variable of light intensity and well-characterized phototransduction cascades make bias-variance analysis straightforward, e.g. using results of Ref. \cite{grzywacz1992response}; and hair cells of the auditory and vestibular systems, where the ordinal structure of frequency tuning makes bias and variance analyses a natural fit \cite{barrett1978transfer}. Beyond individual receptors, the spin-glass MaxEnt framework applied here to cultured cortical neurons could in principle be extended to \textit{in vivo} recordings, to other brain regions, or to other organisms entirely, provided that partition function computation is handled via contrastive divergence or related methods. More broadly, any engineered or evolved sensor system — including those in synthetic biology or neuromorphic computing — for which a generative model of sensor-stimulus relationships can be fit stands to benefit from this style of analysis.}*

6- Several figures contain numerous curves and annotations; increasing the font size of axis labels and legends would improve readability.

*Thank you for the feedback. We have increased font sizes to the largest possible without interfering with data presentation.*

7- The Conclusion could be strengthened by highlighting the broader impact and possible future research directions of this framework.

*Added:*

*\textcolor{blue}{More broadly, this framework represents a general-purpose toolkit for quantifying sensory encoding in any biological system admitting a statistical mechanical or stimulus-dependent Maximum Entropy model. The examples analyzed here - genetic regulatory circuits, ligand-gated ion channels, chemotactic receptors, and cultured neural networks - span several orders of magnitude in biological complexity, suggesting that bias, variance, and confusion matrices may serve as a unifying language for comparing sensory performance across systems and organisms. Future work could extend this framework in several directions: applying it to \textit{in vivo} neural recordings where naturalistic stimulus statistics are available and can replace the uniform prior; developing efficient parameter estimation via contrastive divergence or Minimum Probability Flow to enable analysis of larger neural populations; and combining bias-variance analyses with an understanding of which stimuli it often sees to ask not just how well a sensor performs, but whether its performance is matched to the statistics of its natural environment \cite{barlow1961possible}. The latter direction connects naturally to broader questions in theoretical biology about the degree to which biological sensors are adapted to their ecological niches - questions that the stimulus-resolved metrics introduced here are particularly well suited to answer, adding to information maximization analyses that assume a particular $p(x)$ and derive the information-theoretically optimal $p(s|x)$.}*
