# Supplementary material for: Analyzing an organism’s sensors using Maximum Entropy (MaxEnt) models with bias, variance, and confusion matrices
Source: PLoS One. 2026 Aug 11;21(8):e0342165. doi: 10.1371/journal.pone.0342165 (PMC13460584; doi:10.1371/journal.pone.0342165)
Supplement: Peer review history

# Author comment: Author response to Decision Letter 0

**DOI:** 10.1371/journal.pone.0342165.r001

**Transfer Alert**

This paper was transferred from another journal. As a result, its full editorial history (including decision letters, peer reviews and author responses) may not be present.

16 Jan 2026

# Peer review: Decision Letter 0

**DOI:** 10.1371/journal.pone.0342165.r002

**Authors:** Massimo Mariello

23 Feb 2026

PONE-D-26-02593

Analyzing an organism’s sensors using Maximum Entropy models with bias, variance, and confusion matrices

PLOS One

Dear Dr. Marzen,

Thank you for submitting your manuscript to PLOS ONE. After careful consideration, we feel that it has merit but does not fully meet PLOS ONE’s publication criteria as it currently stands. Therefore, we invite you to submit a revised version of the manuscript that addresses the points raised during the review process.

The manuscript needs more in-depth explanation and justification of results.

Please submit your revised manuscript by Apr 09 2026 11:59PM. If you will need more time than this to complete your revisions, please reply to this message or contact the journal office at plosone@plos.org. When you're ready to submit your revision, log on to https://www.editorialmanager.com/pone/ and select the 'Submissions Needing Revision' folder to locate your manuscript file.

Please include the following items when submitting your revised manuscript:

- A letter that responds to each point raised by the academic editor and reviewer(s). You should upload this letter as a separate file labeled 'Response to Reviewers'.
- A marked-up copy of your manuscript that highlights changes made to the original version. You should upload this as a separate file labeled 'Revised Manuscript with Track Changes'.
- An unmarked version of your revised paper without tracked changes. You should upload this as a separate file labeled 'Manuscript'.

If you would like to make changes to your financial disclosure, please include your updated statement in your cover letter. Guidelines for resubmitting your figure files are available below the reviewer comments at the end of this letter.

If applicable, we recommend that you deposit your laboratory protocols in protocols.io to enhance the reproducibility of your results. Protocols.io assigns your protocol its own identifier (DOI) so that it can be cited independently in the future. For instructions see: https://journals.plos.org/plosone/s/submission-guidelines#loc-laboratory-protocols. Additionally, PLOS ONE offers an option for publishing peer-reviewed Lab Protocol articles, which describe protocols hosted on protocols.io. Read more information on sharing protocols at https://plos.org/protocols?utm_medium=editorial-email&utm_source=authorletters&utm_campaign=protocols.

We look forward to receiving your revised manuscript.

Kind regards,

Massimo Mariello

Academic Editor

PLOS One

Journal Requirements:

When submitting your revision, we need you to address these additional requirements.

1. Please ensure that your manuscript meets PLOS ONE's style requirements, including those for file naming. The PLOS ONE style templates can be found at

https://journals.plos.org/plosone/s/file?id=wjVg/PLOSOne_formatting_sample_main_body.pdf and

https://journals.plos.org/plosone/s/file?id=ba62/PLOSOne_formatting_sample_title_authors_affiliations.pdf

2.  Please note that PLOS One has specific guidelines on code sharing for submissions in which author-generated code underpins the findings in the manuscript. In these cases, we expect all author-generated code to be made available without restrictions upon publication of the work. Please review our guidelines at https://journals.plos.org/plosone/s/materials-and-software-sharing#loc-sharing-code and ensure that your code is shared in a way that follows best practice and facilitates reproducibility and reuse.

3. Thank you for stating the following financial disclosure:

“This work was performed in part at the Aspen Center for Physics and was supported by a grant from the Alfred P. Sloan Foundation (G-2024-22395). Many thanks to the Pioneer Academy for support and hospitality. This study was supported by the US Air Force Office for Scientific Research, Grant Number FA9550-19-1-0411. Research was sponsored by the Army Research Office and was accomplished under Grant Number W911NF-25-1-0260. The views and conclusions contained in this document are those of the authors and should not be interpreted as representing the official policies, either expressed or implied, of the Army Research Office or the U.S. Government. The U.S. Government is authorized to reproduce and distribute reprints for Government purposes notwithstanding any copyright notation herein.”

Please state what role the funders took in the study. If the funders had no role, please state: "The funders had no role in study design, data collection and analysis, decision to publish, or preparation of the manuscript."

If this statement is not correct you must amend it as needed.

Please include this amended Role of Funder statement in your cover letter; we will change the online submission form on your behalf.

4. We note that the grant information you provided in the ‘Funding Information’ and ‘Financial Disclosure’ sections do not match.

When you resubmit, please ensure that you provide the correct grant numbers for the awards you received for your study in the ‘Funding Information’ section.

5. Thank you for stating the following in the Acknowledgments Section of your manuscript:

“This work was performed in part at the Aspen Center for Physics and was supported by a grant from the Alfred P. Sloan Foundation (G-2024-22395). Many thanks to the Pioneer Academy for support and hospitality. This study was supported by the US Air Force Office for Scientific Research, Grant Number FA9550-19-1-0411. Research was sponsored by the Army Research Office and was accomplished under Grant Number W911NF-25-1-0260. The views and conclusions contained in this document are those of the authors and should not be interpreted as representing the official policies, either expressed or implied, of the Army Research Office or the U.S. Government. The U.S.  Government is authorized to reproduce and distribute reprints for Government purposes notwithstanding any copyright notation herein.”

We note that you have provided funding information that is not currently declared in your Funding Statement. However, funding information should not appear in the Acknowledgments section or other areas of your manuscript. We will only publish funding information present in the Funding Statement section of the online submission form.

Please remove any funding-related text from the manuscript and let us know how you would like to update your Funding Statement. Currently, your Funding Statement reads as follows:

“This work was performed in part at the Aspen Center for Physics and was supported by a grant from the Alfred P. Sloan Foundation (G-2024-22395). Many thanks to the Pioneer Academy for support and hospitality. This study was supported by the US Air Force Office for Scientific Research, Grant Number FA9550-19-1-0411. Research was sponsored by the Army Research Office and was accomplished under Grant Number W911NF-25-1-0260. The views and conclusions contained in this document are those of the authors and should not be interpreted as representing the official policies, either expressed or implied, of the Army Research Office or the U.S. Government. The U.S. Government is authorized to reproduce and distribute reprints for Government purposes notwithstanding any copyright notation herein.”

Please include your amended statements within your cover letter; we will change the online submission form on your behalf.

6. Please upload a copy of Supporting Information Figure/Table/etc. “S1 Code Repository. “ which you refer to in your text on page 15.

7. If the reviewer comments include a recommendation to cite specific previously published works, please review and evaluate these publications to determine whether they are relevant and should be cited. There is no requirement to cite these works unless the editor has indicated otherwise.

[Note: HTML markup is below. Please do not edit.]

Reviewers' comments:

Reviewer's Responses to Questions

**Comments to the Author**

1. Is the manuscript technically sound, and do the data support the conclusions?

The manuscript must describe a technically sound piece of scientific research with data that supports the conclusions. Experiments must have been conducted rigorously, with appropriate controls, replication, and sample sizes. The conclusions must be drawn appropriately based on the data presented.

Reviewer #1: Partly

Reviewer #2: Yes

Reviewer #3: Partly

Reviewer #4: Partly

**********

2. Has the statistical analysis been performed appropriately and rigorously?

Reviewer #1: No

Reviewer #2: Yes

Reviewer #3: No

Reviewer #4: No

**********

3. Have the authors made all data underlying the findings in their manuscript fully available?

The PLOS Data policy requires authors to make all data underlying the findings described in their manuscript fully available without restriction, with rare exception (please refer to the Data Availability Statement in the manuscript PDF file). The data should be provided as part of the manuscript or its supporting information, or deposited to a public repository. For example, in addition to summary statistics, the data points behind means, medians and variance measures should be available. If there are restrictions on publicly sharing data—e.g. participant privacy or use of data from a third party—those must be specified.

Reviewer #1: Yes

Reviewer #2: Yes

Reviewer #3: No

Reviewer #4: Yes

**********

4. Is the manuscript presented in an intelligible fashion and written in standard English?

PLOS ONE does not copyedit accepted manuscripts, so the language in submitted articles must be clear, correct, and unambiguous. Any typographical or grammatical errors should be corrected at revision, so please note any specific errors here.

Reviewer #1: Yes

Reviewer #2: Yes

Reviewer #3: Yes

Reviewer #4: Yes

**********

5. Review Comments to the Author

Please use the space provided to explain your answers to the questions above. You may also include additional comments for the author, including concerns about dual publication, research ethics, or publication ethics. (Please upload your review as an attachment if it exceeds 20,000 characters)

Reviewer #1: Review of the Manuscript (PONE-D-26-02593) titled

Analyzing an organism's sensors using Maximum Entropy models with bias, variance, and confusion matrices

This manuscript proposes an alternative framework to mutual information (MI) for analyzing biological sensors, using stimulus-dependent Maximum Entropy (MaxEnt) models combined with maximum likelihood estimation to derive estimators of environmental states. These estimators are then evaluated using bias/variance metrics (for ordinal stimuli) or confusion matrices (for categorical stimuli). The authors apply this approach to ligand-receptor binding models and cultured neuronal networks. The manuscript in its current form suffers from significant methodological and statistical weaknesses that must be checked prior to publication.

• Insufficient statistical justification for replacing mutual information with bias/variance and confusion matrices, without adequately addressing their own estimation biases or undersampling issues.

• The method still faces exponential complexity in partition function calculation, limiting scalability, and relies on ad hoc neuron subset selection.

• Methodologically weak subset selection based only on Pearson correlation, which fails to capture multivariate or dynamic predictive relationships and likely explains discrepancies with prior predictive findings.

• No correction for multiple comparisons, incomplete handling of class imbalance in confusion matrices, and insufficient comparison with mutual information benchmarks.

• Inadequate treatment of temporal dependence due to binning neural activity into independent windows, ignoring autocorrelation that could violate model assumptions.

• Superficial biological interpretation of results without clear mechanistic or statistical explanations linking model outputs to biological function.

Reviewer #2: A little observation about the confidence levels used : statisticians would prefer a 95% or 99% Confidence Interval to 90% confidence interval, as used in the analyses. I would suggest, since a significance level of 5% was used for the Shapiro-Wilk test and for consistency, that 95% confidence intervals be constructed instead of the 90%.

If possible Author(s) should compare their new method with earlier (existing) methods with empirical results before concluding their new method is superior.

Reviewer #3: Suggestions

• More mathematical clarity is required

• Add numerical comparison of MI vs Bias, Variance and confusion matrix results

• Disagreement of results between the two strategies

• Adding effect sizes, confidence intervals, power analysis, etc. can improve clarity

• Cross validation confusion matrices can be useful

• Add full pipeline description, selection and training of the model, tuning of hyperparameters, etc. to make results reproduceable.

• Notations and symbols need to be more consistent

Final Comments

Claim is very strong but without quantification.

Reviewer #4: Journal: PLOS ONE

MS ID: PONE-D-26-02593

Title: Analyzing an organism’s sensors using Maximum Entropy models with bias, variance,

and confusion matrices

Version: 1 Date: 16 February 2026

Reviewer's report:

The manuscript proposes an alternative framework to mutual information for analyzing the performance of biological sensors. The authors argue that while mutual information provides a single, often computationally intractable summary statistic, their method—which combines stimulus-dependent Maximum Entropy (MaxEnt) models with maximum likelihood estimation—offers a more detailed and computationally efficient picture. However, the manuscript has several issues that must be addressed before it can be considered for publication.

Comment 1—The authors' claim of their method being “computationally efficient” is contradicted by their own text, indicating that the assertion of computational tractability is overstated. They have an obligation to demonstrate and quantify that efficiency. Currently, they do not. They state that mutual information is “computationally intractable” (Abstract) and “notoriously difficult to estimate” (Conclusion). They later admit to facing significant “computational difficulties” when applying their MaxEnt model (Conclusion). They even note that calculating the partition function “scales exponentially with system size”. The paper presents a paradox that a scalability study would resolve.

Comment 2—The authors state: “On the condition of normality, t-tests were used to determine the significance of differences”. The phrase “t-tests were used” is insufficient because there are several varieties of t-tests, each answering a different question and having different assumptions. Even if they specify “independent samples,” there is a second layer of ambiguity: Which version of the independent t-test (Student's/Welch's t-test)?

Comment 3—p is used twice in Equation (7), which is slightly confusing. While clear in context, a different symbol (e.g., p0) might improve readability.

Comment 4—Equation 11 holds mathematically. However, if the model is simply fitting the joint distribution of two variables without explicitly handling the time dimension in the probability structure (i.e., treating them as simultaneous events), then using this joint probability for a time-shifted argmax might be statistically valid but biologically misaligned.

Comment 5—The paper should provide a direct, side-by-side comparison of their method to the MI on the same datasets. It would be straightforward to calculate the mutual information and compare it to the bias/variance plots. This would powerfully illustrate what information is "lost" or "gained" by using the more detailed metrics. How do the confusion matrix metrics relate to the MI values? Does a high true positive rate correlate with high MI? Such a comparison would ground the new method and demonstrate its added value concretely.

Comment 6—Pearson correlation assumes linearity and normality. Based on the manuscript's description of the data and the stated goals, is the application of Pearson correlation suitable or problematic?

Comment 7—The statement about a 1.31% improvement in predictive accuracy. While statistically significant, is this improvement biologically or practically meaningful, given that both are near 99%? A brief comment on effect size would be useful.

**********

6. PLOS authors have the option to publish the peer review history of their article (what does this mean?). If published, this will include your full peer review and any attached files.

If you choose “no”, your identity will remain anonymous but your review may still be made public.

**Do you want your identity to be public for this peer review?**  For information about this choice, including consent withdrawal, please see our Privacy Policy.

Reviewer #1: No

Reviewer #2: No

Reviewer #3: **Yes:** Prof. Dr. Tanvir Ahmad

Reviewer #4: **Yes:** Mahmoud A. Abdel-Fattah

**********

[NOTE: If reviewer comments were submitted as an attachment file, they will be attached to this email and accessible via the submission site. Please log into your account, locate the manuscript record, and check for the action link "View Attachments". If this link does not appear, there are no attachment files.]

To ensure your figures meet our technical requirements, please review our figure guidelines: https://journals.plos.org/plosone/s/figures

You may also use PLOS’s free figure tool, NAAS, to help you prepare publication quality figures: https://journals.plos.org/plosone/s/figures#loc-tools-for-figure-preparation.

NAAS will assess whether your figures meet our technical requirements by comparing each figure against our figure specifications.

Attachment Submitted filename: Review.pdf

# Author comment: Author response to Decision Letter 1

**DOI:** 10.1371/journal.pone.0342165.r003

27 Mar 2026

Dear Editor,

Attached is our response to reviewers. Responses are in italics. We hope we have answered all questions and that the article is worthy of review and hopefully publication. Also, our new Funding Statement is as follows:

This work was performed in part at the Aspen Center for Physics and was supported by a grant from the Alfred P. Sloan Foundation (G-2024-22395). Many thanks to the Pioneer Academy for support and hospitality. This study was supported by the US Air Force Office for Scientific Research, Grant Number FA9550-19-1-0411. Research was sponsored by the Army Research Office and was accomplished under Grant Number W911NF-25-1-0260. The views and conclusions contained in this document are those of the authors and should not be interpreted as representing the official policies, either expressed or implied, of the Army Research Office or the U.S. Government. The U.S. Government is authorized to reproduce and distribute reprints for Government purposes notwithstanding any copyright notation herein. The funders had no role in study design, data collection and analysis, decision to publish, or preparation of the manuscript.

Reviewer #1: Review of the Manuscript (PONE-D-26-02593) titled

Analyzing an organism's sensors using Maximum Entropy models with bias, variance, and confusion matrices

This manuscript proposes an alternative framework to mutual information (MI) for analyzing biological sensors, using stimulus-dependent Maximum Entropy (MaxEnt) models combined with maximum likelihood estimation to derive estimators of environmental states. These estimators are then evaluated using bias/variance metrics (for ordinal stimuli) or confusion matrices (for categorical stimuli). The authors apply this approach to ligand-receptor binding models and cultured neuronal networks. The manuscript in its current form suffers from significant methodological and statistical weaknesses that must be checked prior to publication.

We have endeavored to fix these issues.

• Insufficient statistical justification for replacing mutual information with bias/variance and confusion matrices, without adequately addressing their own estimation biases or undersampling issues.

We have added to the Discussion:

\textcolor{blue}{There were two main justifications for using bias and variance or confusion matrices instead of mutual informations. First, bias and variance or confusion matrices provide more information than mutual information, replacing a single number and a hard-to-interpret capacity-achieving distribution in the case that channel capacity is computed with a suite of numbers that describe how precision and accuracy both change as the environmental input changes. Second, estimation of bias and variance or confusion matrices may actually be easier than estimation of notoriously difficult-to-estimate mutual informations.}

\textcolor{blue}{It is worth elaborating upon the statistical advantages of this new method. When statistical mechanical models are used as stimulus-dependent Maximum Entropy models, estimation errors in the bias and variance or confusion matrices result from errors in the statistical mechanical models. These errors cannot be reasonably compared with error in estimating mutual informations, since the errors from statistical mechanical models come from errors in applying underlying physical principles and not estimation errors from limited data. However, in a more comparable case, when data-driven stimulus-dependent Maximum Entropy models are considered, estimating bias and variance or confusion matrices have a slight advantage, even in the limit that the number of data points $N$ is large. The best mutual information estimators have a bias that decays as $1/N$, but a curse of dimensionality causes maintaining accuracy in mutual information estimates to require an exponential growth in $N$ as the dimensionality of the stimulus increases. On the other hand, covariance and mean estimators are typically unbiased and usually only require that $N$ increase as a power law with the dimensionality of the stimulus. Errors in estimating mean and covariance propagate linearly in the small-noise (Gaussian) approximation to errors in estimating Maximum Entropy weights, and thus errors in estimating bias and variance or confusion matrix elements, and so the scalings of errors in bias and variance or confusion matrices are superior to those of mutual informations.}

• The method still faces exponential complexity in partition function calculation, limiting scalability, and relies on ad hoc neuron subset selection.

We have now written in the Discussion:

\textcolor{blue}{This new method may also be advantageous in terms of compute efficiency if state-of-the-art algorithms are employed. Statistical mechanical models, if they have energies or dissociation constants that require estimation, can typically be completed with least-squares estimates that fit binding or activity curves to data. Sometimes, these estimates can be quite accurate even with only ten data points because the functional form for binding curves or activity curves dictated by biophysics is so specific. As for the compute-intensive spin-glass Ising models that can be used as stimulus-dependent Maximum Entropy models of neural activity, there may be a compute benefit to using our new method if better parameter estimation methods are employed. We happened to calculate the partition function in a method similar to that of Ref. \cite{lamberti2023prediction}, which required greedily choosing neurons that most increased the predictive accuracy. This method has exponential compute requirements in the number of neurons that you consider adding. However, one can use contrastive divergence \cite{carreira2005contrastive} to greatly ease computation of weights in spin-glass Ising models. Also, further improvements to Minimum Probability Flow \cite{sohldickstein} or Minimum Energy Flow \cite{hillarhopfield} will allow for extremely compute-efficient alternatives to exact calculation of the partition function.}

• Methodologically weak subset selection based only on Pearson correlation, which fails to capture multivariate or dynamic predictive relationships and likely explains discrepancies with prior predictive findings.

We have now fixed this in the Results by utilizing a method similar to that of the PNAS Nexus that used mutual informations. Now, we greedily add neurons that most increase the predictive accuracy. We say this:

\textcolor{blue}{To choose a subset, we greedily add neurons based on how much they contribute to improving predictive accuracy of our estimator, similar to the algorithm used in Ref. \cite{lamberti2023prediction}. Starting with just the stimulus neuron, we first exhaustively iterate over a subset of two neurons from the pool of 59 neurons, choosing the neuron that best improves predictive accuracy. This neuron is appended to the subset, fixed in place, and an exhaustive search is now done with a three-neuron subset by examining each of the remaining 58 options. This process is repeated until the desired subset size is obtained. With this method, subsystem sizes were chosen from three to six neurons.} The MaxEnt model was then fitted to the data, and the confusion matrix \textcolor{blue}{elements} characterizing the estimator's performance are shown in Fig \ref{fig:cm metrics}.

• No correction for multiple comparisons, incomplete handling of class imbalance in confusion matrices, and insufficient comparison with mutual information benchmarks.

We have now used the Bonferroni correction for the multiple comparisons that we did in first comparing controls with the optical and then electrical with the optical, replacing the p of 0.05 with a p of 0.025. We have added in Results:

Statistics of model metrics over experiments were explored to compare optogenetic and electrical trials. Only two of the time-shifts in Fig \ref{fig:cm metrics}, $-100$ and $0$ ms, are relevant: optogenetic and electrical predictive accuracies are not at all distinguishable at other time-shifts. At a time-shift of 0 ms and a subsystem size of six neurons, \textcolor{blue}{the model for electrical stimulation was normally distributed. With $\alpha=0.05$, performing a Shapiro-Wilk normality test on electrically stimulated samples yielded a $p$-value of $p=0.0998$. However, the same test on optogenetic samples yielded a $p$-value of $p=1.13\cdot 10^{-7}$, indicating non-normality. Since the optogenetic stimulation samples are not normally distributed, to compare the location parameters of electrical and optogenetic stimulation, we first check that each distribution is distinct via the Mann-Whitney $U$ test, which is confirmed with a $p$-value of $p=6.16\cdot 10^{-4}$. Using the Hodges-Lehmann estimator, we then find a $1.71\%$ higher predictive accuracy in electrically stimulated samples compared to optogenetically stimulated samples. This is statistically significant even with a Bonferroni correction for multiple comparisons so that the correct significance level is $p = 0.025$. Considering baseline accuracy already lies at about $97.85\%$, this improvement is equivalent to a fivefold decrease in error rates. We can interpret this as follows: a locally concentrated electrical pulse interacts strongly with a subset of the neurons present, while light pulses interact weakly with the whole system; as such, it is significantly easier to predict behavior in electrical stimulation.} Meanwhile, there was no statistically significant difference between globally stimulated cultures and control trials (\textcolor{blue}{$p = 0.319$ using the Mann-Whitney $U$ test}).

\textcolor{blue}{Repeating this statistical analysis at $-100$ ms and again at six neurons, model predictive accuracy outputs were normally distributed for optogenetic stimulation but not for electrical stimulation: Shapiro-Wilk tests revealed $p=1.26\cdot 10^{-4}$ and $p=0.0627$ for electrical and optogenetic stimulation, respectively. The Mann-Whitney $U$ test applied on the optogenetic and electrical model samples indicates similar distributions with $p=0.122$, but optogenetic samples show a statistically significant difference from baseline with $p=0.0214$ using Welch's $t$-test. Interestingly, electrical model samples are almost identical to baseline, with a Mann-Whitney $U$ test $p$ value of $p=1$.}

Class imbalance is now handled:

\textcolor{blue}{To account for class imbalance (the stimulus is much more likely to be inactive than active), we weighted the KL divergence appropriately such that inaccuracies in guessing activity were penalized proportionately. Moreover, we used the \texttt{scipy.minimize} module for model fitting with a custom-designed \texttt{KL} function to account for class imbalance. In the minimization process, we used the \texttt{L-BFGS-B} optimizer method and ran a maximum of 200 iterations or until the difference between successive iterations was less than a set tolerance of $10^{-7}$. Specifics of implementation can be found in Supplementary Information S1.}

• Inadequate treatment of temporal dependence due to binning neural activity into independent windows, ignoring autocorrelation that could violate model assumptions.

This method does not actually ignore autocorrelations, although it seems to be not including multiple time bins in the model. The question is whether or not the model captures the dependencies between the stimulus now and the network activity previously or in the future. The method automatically does that by having covariance matrices that capture the correlations that relate the stimulus now to the network activity in the future or the past. If there are autocorrelations in the network activity, these will automatically affect the covariance matrices for the present stimulus and the network activity in the future. The PNAS Nexus mutual information estimates have a similar underlying logic, as mutual informations were estimated for the stimulus now and the network activity in the future or the past without building a model of the network activity’s autocorrelation by simply calculating mutual informations between the stimulus now and the network activity in the future or the past. Both the PNAS Nexus results and the results here therefore automatically account for these autocorrelations. If one desires to think of neural codewords as being spread over many time bins, then one can include many time bins in the stimulus-dependent Maximum Entropy model, but we did not do that here. That would be an interesting subject of future inquiry. We now write:

\textcolor{blue}{Time-shifting implicitly takes into account temporal autocorrelations in the network state and the stimulus state, as described in Ref. \cite{lamberti2023prediction}. If there are temporal autocorrelations in the network activity, they will automatically allow for potentially reduced correlations between past or future stimulus activity and present network activity. One way to think about this is to notice that we are essentially marginalizing a probability distribution over successive network states and successive stimulus states in order to retain just the probability distribution over past or future stimulus states and the present network state; but this can be just as well accomplished simply by fitting a Maximum Entropy model to past or future stimulus states and present network states without first fitting to successive network states and then marginalizing.}

• Superficial biological interpretation of results without clear mechanistic or statistical explanations linking model outputs to biological function.

The main idea is that bias reflects the accuracy with which an environmental stimulus can be represented and variance represents the precision with which an environmental stimulus can be represented. This is now described in more detail in the Discussion section:

\textcolor{blue}{Although this new method introduces new quantities that may be hard to interpret, there are natural interpretations of bias and variance with biological significance in the context of biosensing. Bias reflects accuracy, and so if an environmental stimulus has low bias, it is estimated accurately; meanwhile, an environmental stimulus with high bias is estimated inaccurately. Variance reflects precision, and so if an environmental stimulus has low variance, it is estimated with high precision; meanwhile, an environmental stimulus with high variance is estimated with low precision. The bias, variance, and mean-squared error as a function of environmental stimulus reveals which environmental stimuli the biosensor is set up to estimate best. Therefore, for example, bacterial chemotactic receptors are excellent at estimating low concentrations of chemoattractant but find it difficult to estimate high concentrations of chemoattractant accurately even though they estimate these high concentrations with confidence. On the other hand, our analyses show that nACh receptors are excellent estimators of both low and high concentrations of neurotransmitter, but relatively bad estimators of middling concentrations of neurotransmitter. And genetic regulatory circuits are better and better estimators of higher and higher concentrations of the number of repressor molecules, which is a direct readout of how much sugar there is in the environment. Meanwhile, the confusion matrices reveal not only predictive accuracies, but more fine-grained metrics that explain how likely the network is to mistake stimulus silence for stimulus activity and vice versa.}

Reviewer #2: A little observation about the confidence levels used : statisticians would prefer a 95% or 99% Confidence Interval to 90% confidence interval, as used in the analyses. I would suggest, since a significance level of 5% was used for the Shapiro-Wilk test and for consistency, that 95% confidence intervals be constructed instead of the 90%.

Thank you! We have now done this.

If possible Author(s) should compare their new method with earlier (existing) methods with empirical results before concluding their new method is superior.

We have now added mutual information analyses of all ligand-receptor binding situations; see all additional Figures. Because data was not available for

Attachment Submitted filename: PLoS One Biosensors Response (1)-2.docx

# Peer review: Decision Letter 1

**DOI:** 10.1371/journal.pone.0342165.r004

**Authors:** Massimo Mariello

27 Apr 2026

PONE-D-26-02593R1

Analyzing an organism’s sensors using Maximum Entropy models with bias, variance, and confusion matrices

PLOS One

Dear Dr. Marzen,

Thank you for submitting your manuscript to PLOS ONE. After careful consideration, we feel that it has merit but does not fully meet PLOS ONE’s publication criteria as it currently stands. Therefore, we invite you to submit a revised version of the manuscript that addresses the points raised during the review process.

Please submit your revised manuscript by Jun 11 2026 11:59PM. If you will need more time than this to complete your revisions, please reply to this message or contact the journal office at plosone@plos.org. When you're ready to submit your revision, log on to https://www.editorialmanager.com/pone/ and select the 'Submissions Needing Revision' folder to locate your manuscript file.

Please include the following items when submitting your revised manuscript:

- A letter that responds to each point raised by the academic editor and reviewer(s). You should upload this letter as a separate file labeled 'Response to Reviewers'.
- A marked-up copy of your manuscript that highlights changes made to the original version. You should upload this as a separate file labeled 'Revised Manuscript with Track Changes'.
- An unmarked version of your revised paper without tracked changes. You should upload this as a separate file labeled 'Manuscript'.

If you would like to make changes to your financial disclosure, please include your updated statement in your cover letter. Guidelines for resubmitting your figure files are available below the reviewer comments at the end of this letter.

If applicable, we recommend that you deposit your laboratory protocols in protocols.io to enhance the reproducibility of your results. Protocols.io assigns your protocol its own identifier (DOI) so that it can be cited independently in the future. For instructions see: https://journals.plos.org/plosone/s/submission-guidelines#loc-laboratory-protocols. Additionally, PLOS ONE offers an option for publishing peer-reviewed Lab Protocol articles, which describe protocols hosted on protocols.io. Read more information on sharing protocols at https://plos.org/protocols?utm_medium=editorial-email&utm_source=authorletters&utm_campaign=protocols.

As the corresponding author, your ORCID iD is verified in the submission system and will appear in the published article. PLOS supports the use of ORCID, and we encourage all coauthors to register for an ORCID iD and use it as well. Please encourage your coauthors to verify their ORCID iD within the submission system before final acceptance, as unverified ORCID iDs will not appear in the published article. *Only*  the individual author can complete the verification step; PLOS staff *cannot*  verify ORCID iDs on behalf of authors.

We look forward to receiving your revised manuscript.

Kind regards,

Massimo Mariello

Academic Editor

PLOS One

Journal Requirements:

If the reviewer comments include a recommendation to cite specific previously published works, please review and evaluate these publications to determine whether they are relevant and should be cited. There is no requirement to cite these works unless the editor has indicated otherwise.

[Note: HTML markup is below. Please do not edit.]

Reviewers' comments:

Reviewer's Responses to Questions

**Comments to the Author**

1. If the authors have adequately addressed your comments raised in a previous round of review and you feel that this manuscript is now acceptable for publication, you may indicate that here to bypass the “Comments to the Author” section, enter your conflict of interest statement in the “Confidential to Editor” section, and submit your "Accept" recommendation.

Reviewer #1: All comments have been addressed

Reviewer #4: (No Response)

**********

2. Is the manuscript technically sound, and do the data support the conclusions?

The manuscript must describe a technically sound piece of scientific research with data that supports the conclusions. Experiments must have been conducted rigorously, with appropriate controls, replication, and sample sizes. The conclusions must be drawn appropriately based on the data presented.

Reviewer #1: Yes

Reviewer #4: (No Response)

**********

3. Has the statistical analysis been performed appropriately and rigorously?

Reviewer #1: Yes

Reviewer #4: (No Response)

**********

4. Have the authors made all data underlying the findings in their manuscript fully available?

The PLOS Data policy requires authors to make all data underlying the findings described in their manuscript fully available without restriction, with rare exception (please refer to the Data Availability Statement in the manuscript PDF file). The data should be provided as part of the manuscript or its supporting information, or deposited to a public repository. For example, in addition to summary statistics, the data points behind means, medians and variance measures should be available. If there are restrictions on publicly sharing data—e.g. participant privacy or use of data from a third party—those must be specified.

Reviewer #1: Yes

Reviewer #4: (No Response)

**********

5. Is the manuscript presented in an intelligible fashion and written in standard English?

PLOS ONE does not copyedit accepted manuscripts, so the language in submitted articles must be clear, correct, and unambiguous. Any typographical or grammatical errors should be corrected at revision, so please note any specific errors here.

Reviewer #1: Yes

Reviewer #4: (No Response)

**********

6. Review Comments to the Author

Please use the space provided to explain your answers to the questions above. You may also include additional comments for the author, including concerns about dual publication, research ethics, or publication ethics. (Please upload your review as an attachment if it exceeds 20,000 characters)

Reviewer #1: The authors have thoroughly addressed all previous concerns. The revised manuscript is significantly improved.

Reviewer #4: Journal: PLOS ONE

MS ID: PONE-D-26-02593R1

Title: Analyzing an organism’s sensors using Maximum Entropy models with bias, variance,

and confusion matrices

Version: R1 Date: 21 April 2026

Reviewer's report:

Comment 1—The authors would need to cite any empirical benchmarks comparing the runtime of their method with that of MI estimators on the same data.

Comment 2—Adding a brief convergence analysis would substantially strengthen the manuscript's credibility, particularly given the authors' emphasis on computational claims.

**********

7. PLOS authors have the option to publish the peer review history of their article (what does this mean?). If published, this will include your full peer review and any attached files.

If you choose “no”, your identity will remain anonymous but your review may still be made public.

**Do you want your identity to be public for this peer review?**  For information about this choice, including consent withdrawal, please see our Privacy Policy.

Reviewer #1: **Yes:** Mohamed Mohamed Ezzat Abd El-Monsef

Reviewer #4: **Yes:** Mahmoud A. Abdel-Fattah

**********

[NOTE: If reviewer comments were submitted as an attachment file, they will be attached to this email and accessible via the submission site. Please log into your account, locate the manuscript record, and check for the action link "View Attachments". If this link does not appear, there are no attachment files.]

To ensure your figures meet our technical requirements, please review our figure guidelines: https://journals.plos.org/plosone/s/figures

You may also use PLOS’s free figure tool, NAAS, to help you prepare publication quality figures: https://journals.plos.org/plosone/s/figures#loc-tools-for-figure-preparation.

NAAS will assess whether your figures meet our technical requirements by comparing each figure against our figure specifications.

# Author comment: Author response to Decision Letter 2

**DOI:** 10.1371/journal.pone.0342165.r005

4 May 2026

Thank you for the chance to revise. All our comments are in italics.

Comments to the Author

1. If the authors have adequately addressed your comments raised in a previous round of review and you feel that this manuscript is now acceptable for publication, you may indicate that here to bypass the “Comments to the Author” section, enter your conflict of interest statement in the “Confidential to Editor” section, and submit your "Accept" recommendation.

Reviewer #1: All comments have been addressed

Reviewer #4: (No Response)

2. Is the manuscript technically sound, and do the data support the conclusions?

The manuscript must describe a technically sound piece of scientific research with data that supports the conclusions. Experiments must have been conducted rigorously, with appropriate controls, replication, and sample sizes. The conclusions must be drawn appropriately based on the data presented.

Reviewer #1: Yes

Reviewer #4: (No Response)

3. Has the statistical analysis been performed appropriately and rigorously?

Reviewer #1: Yes

Reviewer #4: (No Response)

4. Have the authors made all data underlying the findings in their manuscript fully available?

The PLOS Data policy requires authors to make all data underlying the findings described in their manuscript fully available without restriction, with rare exception (please refer to the Data Availability Statement in the manuscript PDF file). The data should be provided as part of the manuscript or its supporting information, or deposited to a public repository. For example, in addition to summary statistics, the data points behind means, medians and variance measures should be available. If there are restrictions on publicly sharing data—e.g. participant privacy or use of data from a third party—those must be specified.

Reviewer #1: Yes

Reviewer #4: (No Response)

5. Is the manuscript presented in an intelligible fashion and written in standard English?

PLOS ONE does not copyedit accepted manuscripts, so the language in submitted articles must be clear, correct, and unambiguous. Any typographical or grammatical errors should be corrected at revision, so please note any specific errors here.

Reviewer #1: Yes

Reviewer #4: (No Response)

6. Review Comments to the Author

Please use the space provided to explain your answers to the questions above. You may also include additional comments for the author, including concerns about dual publication, research ethics, or publication ethics. (Please upload your review as an attachment if it exceeds 20,000 characters)

Reviewer #1: The authors have thoroughly addressed all previous concerns. The revised manuscript is significantly improved.

Thank you.

Reviewer #4: Journal: PLOS ONE

MS ID: PONE-D-26-02593R1

Title: Analyzing an organism’s sensors using Maximum Entropy models with bias, variance,

and confusion matrices

Version: R1 Date: 21 April 2026

Reviewer's report:

Comment 1—The authors would need to cite any empirical benchmarks comparing the runtime of their method with that of MI estimators on the same data.

Most of the asymptotic work is theoretical, so we have cited the appropriate papers now for a comparison in the large data limit. We reserve other limits that are of specialty interest such as when the number of parameters grows as the number of data points increases for future work, as it is beyond the scope of this paper to tackle that question as well. We wrote:

“\textcolor{blue}{Mutual information estimators have a bias and variance that decay as $1/N$, but a curse of dimensionality causes maintaining accuracy in mutual information estimates to require an exponential growth in $N$ as the dimensionality of the stimulus increases because the estimators used are implicitly estimating probability distributions or approximations thereof \cite{paninski2003estimation}. Some more favorable mutual information estimation procedures that operate on different principles may have different convergence properties based on aspects of the data such as the number of coincidences \cite{nemenman2011coincidences} or more severe requirements if the dimensionality of the problem grows with the amount of data \cite{paninski2003estimation}. On the other hand, our estimators may have similar variance scalings, but do not require exponentially large amounts of data as dimensionality of the stimulus increases. And so, the scalings of errors in bias and variance or confusion matrices are superior to those of mutual informations.}”

Comment 2—Adding a brief convergence analysis would substantially strengthen the manuscript's credibility, particularly given the authors' emphasis on computational claims.

We now do this in a new subsection:

“\subsection*{Convergence properties of new biosensor metrics}

\textcolor{blue}{Maximum Entropy parameters are in fact maximum likelihood estimators in that they best fit exponential linear models to data. Hence, they are asymptotically normal and unbiased with variance that goes as $1/N$ \cite{cramer1999mathematical}. Here we offer a sketch of a proof for how that translates to convergence of the estimators of bias, variance, mean-squared error, and confusion matrices. In the limit that the amount of data is large, $1/N$ is quite small, and we are justified in making a small noise approximation in which statistics are roughly Gaussian. In particular, confusion matrix elements or bias, variance, and mean-squared error can be said to vary linearly with the variance of Maximum Entropy parameters via typical error propagation techniques of $\sigma_{Y}^2 = \sum_i \left(\frac{\partial Y}{\partial J_i}\right)^2\sigma_{J_i}^2$, where $J_i$ are Maximum Entropy parameters and $Y$ is the performance metric. As such, $\sigma_Y^2$ asymptotically goes as $1/N$, meaning that the variance in performance metrics goes as $1/N$. To estimate Maximum Entropy parameters $J_i$, we need to populate means and covariances and potentially some higher-order moments, depending on the Maximum Entropy model, which means that we need to estimate a number of elements that increases as a power law in the dimensionality of the stimulus. Therefore, $N$ needs only increase in power law fashion with the dimensionality of the stimulus.}”

Attachment Submitted filename: Response to Reviewers.docx

# Editor report: Decision Letter 2

**DOI:** 10.1371/journal.pone.0342165.r006

**Authors:** Massimo Mariello

27 May 2026

PONE-D-26-02593R2

Analyzing an organism’s sensors using Maximum Entropy models with bias, variance, and confusion matrices

PLOS One

Dear Dr. Marzen,

Thank you for submitting your manuscript to PLOS ONE. After careful consideration, we feel that it has merit but does not fully meet PLOS ONE’s publication criteria as it currently stands. Therefore, we invite you to submit a revised version of the manuscript that addresses the points raised during the review process.

The manuscript needs few more in-depth explanations.

Please submit your revised manuscript by Jul 11 2026 11:59PM. If you will need more time than this to complete your revisions, please reply to this message or contact the journal office at plosone@plos.org. When you're ready to submit your revision, log on to https://www.editorialmanager.com/pone/ and select the 'Submissions Needing Revision' folder to locate your manuscript file.

Please include the following items when submitting your revised manuscript:

- A letter that responds to each point raised by the academic editor and reviewer(s). You should upload this letter as a separate file labeled 'Response to Reviewers'.
- A marked-up copy of your manuscript that highlights changes made to the original version. You should upload this as a separate file labeled 'Revised Manuscript with Track Changes'.
- An unmarked version of your revised paper without tracked changes. You should upload this as a separate file labeled 'Manuscript'.

If you would like to make changes to your financial disclosure, please include your updated statement in your cover letter. Guidelines for resubmitting your figure files are available below the reviewer comments at the end of this letter.

If applicable, we recommend that you deposit your laboratory protocols in protocols.io to enhance the reproducibility of your results. Protocols.io assigns your protocol its own identifier (DOI) so that it can be cited independently in the future. For instructions see: https://journals.plos.org/plosone/s/submission-guidelines#loc-laboratory-protocols. Additionally, PLOS ONE offers an option for publishing peer-reviewed Lab Protocol articles, which describe protocols hosted on protocols.io. Read more information on sharing protocols at https://plos.org/protocols?utm_medium=editorial-email&utm_source=authorletters&utm_campaign=protocols.

As the corresponding author, your ORCID iD is verified in the submission system and will appear in the published article. PLOS supports the use of ORCID, and we encourage all coauthors to register for an ORCID iD and use it as well. Please encourage your coauthors to verify their ORCID iD within the submission system before final acceptance, as unverified ORCID iDs will not appear in the published article. *Only*  the individual author can complete the verification step; PLOS staff *cannot*  verify ORCID iDs on behalf of authors.

We look forward to receiving your revised manuscript.

Kind regards,

Massimo Mariello

Academic Editor

PLOS One

Journal Requirements:

1. If the reviewer comments include a recommendation to cite specific previously published works, please review and evaluate these publications to determine whether they are relevant and should be cited. There is no requirement to cite these works unless the editor has indicated otherwise.

2. Please review your reference list to ensure that it is complete and correct. If you have cited papers that have been retracted, please include the rationale for doing so in the manuscript text, or remove these references and replace them with relevant current references. Any changes to the reference list should be mentioned in the rebuttal letter that accompanies your revised manuscript. If you need to cite a retracted article, indicate the article’s retracted status in the References list and also include a citation and full reference for the retraction notice.

[Note: HTML markup is below. Please do not edit.]

[NOTE: If reviewer comments were submitted as an attachment file, they will be attached to this email and accessible via the submission site. Please log into your account, locate the manuscript record, and check for the action link "View Attachments". If this link does not appear, there are no attachment files.]

To ensure your figures meet our technical requirements, please review our figure guidelines: https://journals.plos.org/plosone/s/figures

You may also use PLOS’s free figure tool, NAAS, to help you prepare publication quality figures: https://journals.plos.org/plosone/s/figures#loc-tools-for-figure-preparation.

NAAS will assess whether your figures meet our technical requirements by comparing each figure against our figure specifications.

# Author comment: Author response to Decision Letter 3

**DOI:** 10.1371/journal.pone.0342165.r007

22 Jun 2026

Dear Editors,

Thank you for the chance to revise. All our comments are in italics.

Comments to the Author from the Editor

“The manuscript needs a few more in-depth explanations.”

We have now added a few more in-depth explanations and are happy to add more explanations as the Editor suggests:

\textcolor{blue}{Stimulus-dependent MaxEnt models are not so common, but there are a plethora of procedures for creating equilibrium statistical mechanical models of biological processes. One might wonder if, in fact, these procedures need to be altered for our method. The answer to this is a simple ``no''. It is, in fact, common for statistical mechanical models of biological processes to implicitly act as conditional distributions that describe how sensor state relates to environmental signal. For instance, if a statistical mechanical model is made of gene regulation, then the probability of RNAP binding depends on concentrations of transcription factors, which in turn relate to environmental signals such as sugar concentration \cite{garcia2011quantitative}. This example is made as explicit is possible in later. As a result, the typical methodology for generating statistical mechanical models from biological cartoons needs no augmentation for the method proposed in this manuscript to be used.}

Once we have $p_\text{model}(x \mid \vb{s})$, we utilize \textcolor{blue}{Maximum a Posteriori estimation} to form an estimator of the environment from the sensor state:

\begin{equation}

\hat{x}(\vb{s}) = \argmax_x p_\text{model}(x \mid \vb{s}).

\end{equation}

If we have a joint MaxEnt model, we can find this as

\begin{equation}

\hat{x}(\vb{s}) = \argmax_x \frac{p_\text{model}(x,\vb{s})}{p_\text{model}(\vb{s})} = \argmax_x p_\text{model}(x,\vb{s}).

\end{equation}

If instead we have $p_\text{model}(\vb{s} \mid x)$ as with the statistical mechanical models, we can find the estimator as

\begin{equation}

\hat{x}(\vb{s}) = \argmax_x \frac{p_\text{model}(\vb{s} \mid x) \, p(x)}{p(\vb{s})} = \argmax_x p_\text{model}(\vb{s} \mid x)\,p(x).

\end{equation}

We can approximate $p(x)$ from data or from another model. For our particular neural system, we use $p(x) = p_\text{data}(x)$. We could instead use an unbiased model of the environment, making $p(x)$ uniform, leading to

\begin{equation}

\hat{x}(\vb{s}) = \argmax_x p_\text{model}(\vb{s} \mid x).

\end{equation}

\textcolor{blue}{This corresponds to Maximum Likelihood Estimation.}

\subsection*{Bias, variance, and mean-squared error with \textcolor{blue}{continuous environmental variables}}

\textcolor{blue}{In other words, the estimators introduced here are consistent and also have a variance that decays as $1/N$ and a bias that decays at most as $1/N$, where $N$ is the amount of data points. This alone does not provide us with a computational benefit over mutual information estimation. However, if we examine how $N$ must change to maintain accuracy as the dimensionality of the data increases, we find an immense computational benefit. With mutual information estimates, $N$ must increase exponentially with the dimensionality with many estimators. The reason for this is that histograms over bins that increase in an exponential fashion with the dimensionality of the environmental input and sensor state are estimated in order to understand mutual information. Meanwhile, for these biosensor metrics here, $N$ need only increase in a power law fashion. For very high-dimensional datasets, confusion matrices have a computational advantage. The only caveat is that sometimes, Maximum Entropy estimation is difficult because the state space grows exponentially with the dimensionality of the environmental input and sensor state, but there are well-known workarounds for this such as contrastive divergence \cite{carreira2005contrastive} that we did not use here.}

Attachment Submitted filename: Response_to_Reviewers_auresp_3.docx

# Peer review: Decision Letter 3

**DOI:** 10.1371/journal.pone.0342165.r008

**Authors:** Massimo Mariello

6 Jul 2026

PONE-D-26-02593R3

Analyzing an organism’s sensors using Maximum Entropy models with bias, variance, and confusion matrices

PLOS One

Dear Dr. Marzen,

Thank you for submitting your manuscript to PLOS ONE. After careful consideration, we feel that it has merit but does not fully meet PLOS ONE’s publication criteria as it currently stands. Therefore, we invite you to submit a revised version of the manuscript that addresses the points raised during the review process.

Please submit your revised manuscript by Aug 20 2026 11:59PM. If you will need more time than this to complete your revisions, please reply to this message or contact the journal office at plosone@plos.org. When you're ready to submit your revision, log on to https://www.editorialmanager.com/pone/ and select the 'Submissions Needing Revision' folder to locate your manuscript file.

Please include the following items when submitting your revised manuscript:

- A letter that responds to each point raised by the academic editor and reviewer(s). You should upload this letter as a separate file labeled 'Response to Reviewers'.
- A marked-up copy of your manuscript that highlights changes made to the original version. You should upload this as a separate file labeled 'Revised Manuscript with Track Changes'.
- An unmarked version of your revised paper without tracked changes. You should upload this as a separate file labeled 'Manuscript'.

If you would like to make changes to your financial disclosure, please include your updated statement in your cover letter. Guidelines for resubmitting your figure files are available below the reviewer comments at the end of this letter.

If applicable, we recommend that you deposit your laboratory protocols in protocols.io to enhance the reproducibility of your results. Protocols.io assigns your protocol its own identifier (DOI) so that it can be cited independently in the future. For instructions see: https://journals.plos.org/plosone/s/submission-guidelines#loc-laboratory-protocols. Additionally, PLOS ONE offers an option for publishing peer-reviewed Lab Protocol articles, which describe protocols hosted on protocols.io. Read more information on sharing protocols at https://plos.org/protocols?utm_medium=editorial-email&utm_source=authorletters&utm_campaign=protocols.

As the corresponding author, your ORCID iD is verified in the submission system and will appear in the published article. PLOS supports the use of ORCID, and we encourage all coauthors to register for an ORCID iD and use it as well. Please encourage your coauthors to verify their ORCID iD within the submission system before final acceptance, as unverified ORCID iDs will not appear in the published article. *Only*  the individual author can complete the verification step; PLOS staff *cannot*  verify ORCID iDs on behalf of authors.

We look forward to receiving your revised manuscript.

Kind regards,

Massimo Mariello

Academic Editor

PLOS One

Journal Requirements:

If the reviewer comments include a recommendation to cite specific previously published works, please review and evaluate these publications to determine whether they are relevant and should be cited. There is no requirement to cite these works unless the editor has indicated otherwise.

Please review your reference list to ensure that it is complete and correct. If you have cited papers that have been retracted, please include the rationale for doing so in the manuscript text, or remove these references and replace them with relevant current references. Any changes to the reference list should be mentioned in the rebuttal letter that accompanies your revised manuscript. If you need to cite a retracted article, indicate the article’s retracted status in the References list and also include a citation and full reference for the retraction notice.

[Note: HTML markup is below. Please do not edit.]

Reviewers' comments:

Reviewer's Responses to Questions

**Comments to the Author**

1. If the authors have adequately addressed your comments raised in a previous round of review and you feel that this manuscript is now acceptable for publication, you may indicate that here to bypass the “Comments to the Author” section, enter your conflict of interest statement in the “Confidential to Editor” section, and submit your "Accept" recommendation.

Reviewer #4: All comments have been addressed

Reviewer #5: All comments have been addressed

**********

2. Is the manuscript technically sound, and do the data support the conclusions?

The manuscript must describe a technically sound piece of scientific research with data that supports the conclusions. Experiments must have been conducted rigorously, with appropriate controls, replication, and sample sizes. The conclusions must be drawn appropriately based on the data presented.

Reviewer #4: Yes

Reviewer #5: Yes

**********

3. Has the statistical analysis been performed appropriately and rigorously?

Reviewer #4: Yes

Reviewer #5: N/A

**********

4. Have the authors made all data underlying the findings in their manuscript fully available?

The PLOS Data policy requires authors to make all data underlying the findings described in their manuscript fully available without restriction, with rare exception (please refer to the Data Availability Statement in the manuscript PDF file). The data should be provided as part of the manuscript or its supporting information, or deposited to a public repository. For example, in addition to summary statistics, the data points behind means, medians and variance measures should be available. If there are restrictions on publicly sharing data—e.g. participant privacy or use of data from a third party—those must be specified.

Reviewer #4: Yes

Reviewer #5: Yes

**********

5. Is the manuscript presented in an intelligible fashion and written in standard English?

PLOS ONE does not copyedit accepted manuscripts, so the language in submitted articles must be clear, correct, and unambiguous. Any typographical or grammatical errors should be corrected at revision, so please note any specific errors here.

Reviewer #4: Yes

Reviewer #5: Yes

**********

6. Review Comments to the Author

Please use the space provided to explain your answers to the questions above. You may also include additional comments for the author, including concerns about dual publication, research ethics, or publication ethics. (Please upload your review as an attachment if it exceeds 20,000 characters)

Reviewer #4: Journal: PLOS ONE

MS ID: PONE-D-26-02593R3

Title: Analyzing an organism’s sensors using Maximum Entropy models with bias, variance, and confusion matrices

Version: R3 Date: 01 July 2026

Reviewer's report:

The authors have addressed the major concerns raised in prior reviews across three revision rounds. The manuscript is now ready for publication. The discussion of computational limitations enhances the manuscript's credibility. Remaining minor typographical issues may be handled during the proofreading stage.

Reviewer #5: 1- The manuscript is scientifically sound, and the proposed methodology is novel and relevant to computational biology and statistical physics.

2- The justification for replacing reciprocal information with bias, variance, and confusion matrices is compelling; nonetheless, the merits and drawbacks of the proposed framework may be summarised more explicitly in the Discussion or Conclusion.

3- The suggested method's computational complexity for large-scale biological networks should be briefly discussed by the authors, along with its practical scalability.

4-Some mathematical symbols and notation should be defined immediately when first introduced to improve readability for non-specialist readers.

5- In the Discussion, the biological implications of the results might be further highlighted, especially with regard to possible applications to other sensory systems outside of the examples given.

6- Several figures contain numerous curves and annotations; increasing the font size of axis labels and legends would improve readability.

7- The Conclusion could be strengthened by highlighting the broader impact and possible future research directions of this framework.

**********

7. PLOS authors have the option to publish the peer review history of their article (what does this mean?). If published, this will include your full peer review and any attached files.

If you choose “no”, your identity will remain anonymous but your review may still be made public.

**Do you want your identity to be public for this peer review?**  For information about this choice, including consent withdrawal, please see our Privacy Policy.

Reviewer #4: **Yes:** Mahmoud A. Abdel-Fattah

Reviewer #5: No

**********

[NOTE: If reviewer comments were submitted as an attachment file, they will be attached to this email and accessible via the submission site. Please log into your account, locate the manuscript record, and check for the action link "View Attachments". If this link does not appear, there are no attachment files.]

To ensure your figures meet our technical requirements, please review our figure guidelines: https://journals.plos.org/plosone/s/figures

You may also use PLOS’s free figure tool, NAAS, to help you prepare publication quality figures: https://journals.plos.org/plosone/s/figures#loc-tools-for-figure-preparation.

NAAS will assess whether your figures meet our technical requirements by comparing each figure against our figure specifications.

# Author comment: Author response to Decision Letter 4

**DOI:** 10.1371/journal.pone.0342165.r009

15 Jul 2026

Dear Editor,

Thank you for another round of review. We have responded to all queries from the reviewer, as well as sending our paper to a colleague and clarifying additional details based on his comments. We hope the paper is finally worthy of acceptance!

Our comments are in italics and reviewer comments are in regular font below.

Sincerely,

Chris Wang and Sarah Marzen

Reviewer #5: 1- The manuscript is scientifically sound, and the proposed methodology is novel and relevant to computational biology and statistical physics.

Thank you so much. We tried to use your additional comments to improve the manuscript.

2- The justification for replacing reciprocal information with bias, variance, and confusion matrices is compelling; nonetheless, the merits and drawbacks of the proposed framework may be summarised more explicitly in the Discussion or Conclusion.

See Table 2 in the Conclusions now that summarizes the benefits and drawbacks of the two methods, reproduced here:

\begin{table}[h]

\centering

\caption{\textcolor{blue}{Comparison of mutual information, bias/variance/MSE, and confusion matrix approaches to biosensor analysis.}}

\label{tab:comparison}

\begin{tabular}{p{2.9cm}p{2.9cm}p{2.9cm}p{2.9cm}}

& \textbf{Mutual Information} & \textbf{Bias/Variance} & \textbf{Confusion Matrix} \\

\textbf{Variable type}

& Any

& Ordinal

& Categorical \\[6pt]

\textbf{Output}

& Single scalar

& Function of stimulus value

& $n^2 - n$ off-diagonal elements \\[6pt]

\textbf{What it reveals}

& Total information shared

& Accuracy \& precision at each stimulus level

& Error type at each stimulus category \\[6pt]

\textbf{Computational scaling with data $N$}

& Exponential in stimulus dimensionality

& Power law in stimulus dimensionality

& Power law in stimulus dimensionality \\[6pt]

\textbf{Requires $p(x)$?}

& No

& Only for MAP; MLE does not

& Only for MAP; MLE does not \\[6pt]

\textbf{Interpretability}

& Abstract

& Intuitive (bias $=$ accuracy, variance $=$ precision)

& Intuitive (true positive/negative rates) \\[6pt]

\textbf{Main limitation}

& Undersampling bias; one number hides stimulus-specific structure

& Requires parametric sensor model

& Exponential partition function for high-dimensional $\mathbf{s}$ \\

\end{tabular}

\end{table}

3- The suggested method's computational complexity for large-scale biological networks should be briefly discussed by the authors, along with its practical scalability.

Added:

\textcolor{blue}{This new method compares favorably to mutual information estimation in terms of computational complexity. When statistical mechanical models are known \textit{a priori}, parameter estimation reduces to a standard least-squares fit of binding or activity curves — sometimes requiring as few as ten data points, given the specificity of biophysically-derived functional forms. For data-driven Maximum Entropy models, the primary bottleneck is partition function computation: In our neural network application, we computed the partition function using a greedy neuron selection procedure similar to Ref.~\cite{lamberti2023prediction}, whose computational requirements grow faster than exponentially in the number of candidate neurons. However, estimation of coupling constants in spin-glass Ising models is a well-studied problem with efficient solutions. Contrastive divergence \cite{carreira2005contrastive} greatly eases computation of spin-glass weights, and Minimum Probability Flow \cite{sohldickstein} or Minimum Energy Flow \cite{hillarhopfield} provide extremely compute-efficient alternatives to exact partition function calculation. Adoption of these methods would allow the framework to scale to much larger neural populations while retaining the interpretive advantages demonstrated here.} Also, further improvements to Minimum Probability Flow \cite{sohldickstein} or Minimum Energy Flow \cite{hillarhopfield} will allow for extremely compute-efficient alternatives to exact calculation of the partition function.

4-Some mathematical symbols and notation should be defined immediately when first introduced to improve readability for non-specialist readers.

Thank you for the clarification request. We have gone back and added the following to best clarify possibly confusing symbols and notation:

1.

\begin{equation}\textcolor{blue}{

p_{\mathrm{model}}(x, \vb{s}) = \frac{1}{Z} \exp\left(-E_{\theta}(x, \vb{s})\right)}

\end{equation}

\textcolor{blue}{where $Z$ is the partition function, or the sum of $\exp(-E_\theta(x,\vb{s}))$ over all values of $x$ and $\vb{s}$, which serves as a normalization factor. Assuming the distribution is constrained and parametrized by a set of parameters $\theta$, we aim to} fit the data distribution…

2.

\textcolor{blue}{Here, $J$ is decomposed into four block matrices. Assuming a system size of $n$, $J_{xx}$ is a scalar interpreted as the stimulus firing propensity, $J_{sx} = J_{xs}^\top$ are $n-1$-dimensional vectors that represent stimulus-network couplings, and $J_{ss}$ is an $n-1\times n-1$ matrix describing the couplings between network elements.}

3.

The output of the statistical mechanical model \cite{garcia2011quantitative} is the following model for $p_\text{model}(s \mid x)$, where $s$ is whether or not RNAP is bound and $x$ is the number of \textcolor{blue}{Lac repressor (lacR) tetramers:

\begin{equation}

p_\text{model}(s \mid x) = \frac{p_0}{1+p_0+\frac{x}{N_\text{NS}}\exp(-\beta\Delta\epsilon_\text{rd})}.

\end{equation}

In the case of a single sensor, we may switch from the boldfaced $\vb{s}$ to $s$ to denote sensor state. Here, $p_0$ is the number of RNAP molecules, $P$, divided by the number of nonspecific DNA sites, $N_\mathrm{NS}$, with values of $p_0\approx 10^{-3}$ and $N_\text{NS}\approx 5\times 10^6$, and $\Delta\epsilon_\text{rd}$ is the repressor-DNA binding energy difference, which adopts a range of values that can be tuned in experiments \cite{garcia2011quantitative}. Finally, $\beta=1/k_B T$, the inverse temperature.}

4.

\textcolor{blue}{To briefly contextualize: $x$ is the ligand concentration, $K$ is the dissociation constant, $\Delta \epsilon$ is the cooperativity energy, and $\beta = 1/k_B T$.}

5- In the Discussion, the biological implications of the results might be further highlighted, especially with regard to possible applications to other sensory systems outside of the examples given.

Added:

\textcolor{blue}{The framework introduced here is broadly applicable beyond the specific biosensors analyzed. Any biological system whose response can be modeled as a conditional distribution $p(s∣x)$, even if it maps input time series to output time series, is immediately amenable to this analysis. Other sensory systems that could be studied using these methods include: olfactory receptors, whose dose-response curves are well-described by MWC-type models \cite{martins2011trade} and for which bias and variance as a function of odorant concentration would clarify which odors are encoded reliably; photoreceptors, where the ordinal variable of light intensity and well-characterized phototransduction cascades make bias-variance analysis straightforward, e.g. using results of Ref. \cite{grzywacz1992response}; and hair cells of the auditory and vestibular systems, where the ordinal structure of frequency tuning makes bias and variance analyses a natural fit \cite{barrett1978transfer}. Beyond individual receptors, the spin-glass MaxEnt framework applied here to cultured cortical neurons could in principle be extended to \textit{in vivo} recordings, to other brain regions, or to other organisms entirely, provided that partition function computation is handled via contrastive divergence or related methods. More broadly, any engineered or evolved sensor system — including those in synthetic biology or neuromorphic computing — for which a generative model of sensor-stimulus relationships can be fit stands to benefit from this style of analysis.}

6- Several figures contain numerous curves and annotations; increasing the font size of axis labels and legends would improve readability.

Thank you for the feedback. We have increased font sizes to the largest possible without interfering with data presentation.

7- The Conclusion could be strengthened by highlighting the broader impact and possible future research directions of this framework.

Added:

\textcolor{blue}{More broadly, this framework represents a general-purpose toolkit for quantifying sensory encoding in any biological system admitting a statistical mechanical or stimulus-dependent Maximum Entropy model. The examples analyzed here - genetic regulatory circuits, ligand-gated ion channels, chemotactic receptors, and cultured neural networks - span several orders of magnitude in biological complexity, suggesting that bias, variance, and confusion matrices may serve as a unifying language for comparing sensory performance across systems and organisms. Future work could extend this framework in several directions: applying it to \textit{in vivo} neural recordings where naturalistic stimulus statistics are available and can replace the uniform prior; developing efficient parameter estimation via contrastive divergence or Minimum Probability Flow to enable analysis of larger neural populations; and combining bias-variance analyses with an understanding of which stimuli it often sees to ask not just how well a sensor performs, but whether its performance is matched to the statistics of its natural environment \cite{barlow1961possible}. The latter direction connects naturally to broader questions in theoretical biology about the degree to which biological sensors are adapted to their ecological niches - questions that the stimulus-resolved metrics introduced here are particularly well suited to answer, adding to information maximization analyses that assume a particular $p(x)$ and derive the information-theoretically optimal $p

Attachment Submitted filename: Response to referees 77.docx

# Editor report: Decision Letter 4

**DOI:** 10.1371/journal.pone.0342165.r010

**Authors:** Massimo Mariello

20 Jul 2026

Analyzing an organism’s sensors using Maximum Entropy models with bias, variance, and confusion matrices

PONE-D-26-02593R4

Dear Dr. Marzen,

We’re pleased to inform you that your manuscript has been judged scientifically suitable for publication and will be formally accepted for publication once it meets all outstanding technical requirements.

Within one week, you’ll receive an e-mail detailing the required amendments. When these have been addressed, you’ll receive a formal acceptance letter and your manuscript will be scheduled for publication.

An invoice will be generated when your article is formally accepted. Please note, if your institution has a publishing partnership with PLOS and your article meets the relevant criteria, all or part of your publication costs will be covered. Please make sure your user information is up-to-date by logging into Editorial Manager at Editorial Manager® and clicking the ‘Update My Information' link at the top of the page. For questions related to billing, please contact billing support.

If your institution or institutions have a press office, please notify them about your upcoming paper to help maximize its impact. If they’ll be preparing press materials, please inform our press team as soon as possible -- no later than 48 hours after receiving the formal acceptance. Your manuscript will remain under strict press embargo until 2 pm Eastern Time on the date of publication. For more information, please contact onepress@plos.org.

Kind regards,

Massimo Mariello

Academic Editor

PLOS One

Additional Editor Comments (optional):

Reviewers' comments:

# Editor report: Acceptance letter

**DOI:** 10.1371/journal.pone.0342165.r011

**Authors:** Massimo Mariello

PONE-D-26-02593R4

PLOS One

Dear Dr. Marzen,

I'm pleased to inform you that your manuscript has been deemed suitable for publication in PLOS One. Congratulations! Your manuscript is now being handed over to our production team.

At this stage, our production department will prepare your paper for publication. This includes ensuring the following:

* All references, tables, and figures are properly cited

* All relevant supporting information is included in the manuscript submission,

* There are no issues that prevent the paper from being properly typeset

You will receive further instructions from the production team, including instructions on how to review your proof when it is ready. Please keep in mind that we are working through a large volume of accepted articles, so please give us a few days to review your paper and let you know the next and final steps.

Lastly, if your institution or institutions have a press office, please let them know about your upcoming paper now to help maximize its impact. If they'll be preparing press materials, please inform our press team within the next 48 hours. Your manuscript will remain under strict press embargo until 2 pm Eastern Time on the date of publication. For more information, please contact onepress@plos.org.

You will receive an invoice from PLOS for your publication fee after your manuscript has reached the completed accept phase. If you receive an email requesting payment before acceptance or for any other service, this may be a phishing scheme. Learn how to identify phishing emails and protect your accounts at https://explore.plos.org/phishing.

If we can help with anything else, please email us at customercare@plos.org.

Thank you for submitting your work to PLOS ONE and supporting open access.

Kind regards,

PLOS ONE Editorial Office Staff

on behalf of

Dr. Massimo Mariello

Academic Editor

PLOS One
